# Reduced synaptic vesicle protein 2A in extracellular vesicles and brains of Alzheimer’s disease: associations with Aβ, tau, synaptic proteins and *APOE* ε4

**DOI:** 10.1186/s40035-025-00508-2

**Published:** 2025-09-24

**Authors:** Jana Nussbaumer, Aatmika Barve, Valentin Zufferey, Jeanne Espourteille, Tunahan Kirabali, Uwe Konietzko, Daniel Razansky, Axel Rominger, Agneta Nordberg, Luc Buée, Morvane Colin, Roger M. Nitsch, Christoph Hock, Kevin Richetin, Ruiqing Ni

**Affiliations:** 1 Institute for Regenerative Medicine, University of Zurich, Wagistrasse 12, 8952 Schlieren, Switzerland; 2 Department of Psychiatry, Center for Psychiatric Neurosciences, Lausanne University Hospital (CHUV) and University of Lausanne, 1008 Prilly-Lausanne, Switzerland; 3 Institute for Biomedical Engineering, University of Zurich and ETH Zurich, WolfgangPauli strasse 27, 8093 Zurich, Switzerland; 4 Department of Nuclear Medicine, Inselspital University of Bern, Freiburgstrasse 18, 3010 Bern, Switzerland; 5 Division of Clinical Geriatrics, Center for Alzheimer Research, Department of Neurobiology, Care Sciences and Society, Karolinska Institute, Stockholm, Sweden; 6 Univ. Lille, Inserm, CHU Lille, LilNCog-Lille Neuroscience and Cognition, Lille, France; 7 Neurimmune, Schlieren, Switzerland; 8 Department of Clinal Neurosciences, Leenaards Memory Center, Lausanne University Hospital (CHUV) and University of Lausanne, Chem. de Mont-Paisible 16, 1011 Lausanne, Switzerland

**Keywords:** Amyloid-β, Alzheimer’s disease, *APOE* ε4, Extracellular vesicles, Proteomics, Synaptic vesicle protein 2A, Synaptophysin, Tau

## Abstract

**Background:**

Alzheimer’s disease (AD) is characterized by accumulation of amyloid-β (Aβ) plaques, tau neurofibrillary Tangles and synaptic dysfunction. The aim of this study was to map the distributions of synaptic vesicle protein 2A (SV2A) and other synaptic proteins in the brain and the brain-derived extracellular vesicles (BDEVs) of AD patients, analyze their associations with Aβ, tau, and the apolipoprotein E (*APOE*) ε4 allele, and investigate the biological role of SV2A.

**Methods:**

Mass spectrometry-based proteomics of BDEVs and immunohistochemistry staining were conducted on postmortem brain samples from 57 AD patients and 48 nondemented controls. The levels of SV2A, synaptophysin (SYP), and other synaptic proteins in the brain tissues and the BDEVs, and their associations with Aβ, tau (phospho-tau and Braak stages), other proteins and the *APOE* ε4 allele, were analyzed.

**Results:**

SV2A levels were significantly lower in AD patients than in nondemented controls, particularly in the hippocampus and entorhinal cortex. *APOE* ε4 carriers presented further reductions in SV2A levels compared with noncarriers. The SV2A levels in BDEVs and brain tissues were positively correlated with SYP levels and negatively correlated with Aβ and phospho-tau levels. Reductions in SV2A were associated with decreased levels of other synaptic proteins, such as synaptotagmins, GAP43, and SNAP25. SV2A emerged as a central hub with interactions with proteins from subnetworks related to synaptic vesicle formation and fusion.

**Conclusion:**

SV2A levels in brain tissues and BDEVs are reduced in AD patients, particularly in those carrying the *APOE* ε4 allele, and are correlated with Aβ and tau pathologies. SV2A may serve as a valuable biomarker for monitoring synaptic dysfunction and progression in AD.

**Supplementary Information:**

The online version contains supplementary material available at 10.1186/s40035-025-00508-2.

## Introduction

Alzheimer’s disease (AD) is a neurodegenerative disorder characterized by progressive memory loss and cognitive decline [1]. AD is pathologically characterized by abnormal accumulation of amyloid-β (Aβ) plaques and neurofibrillary tangles (NFTs) formed by hyperphosphorylated tau [2]. Synaptic damage and loss are downstream effects of amyloidosis and tauopathy and are recognized as among the earliest neurobiological correlates of cognitive impairment in AD [3–6]. Neuropathological studies have shown that the synaptic protein levels are lower in NFT-containing neurons than in those without NFTs, and are correlated with the Braak stage and the level of NFT [7–9]. Pathogenic tau binds to synaptic vesicles, dysregulates the synaptic proteome and interferes with presynaptic functions, synaptic transmission, synaptic vesicle mobility and the release rate, leading to synaptic loss [10–13]. Treatments targeting synaptic dysfunction via synaptic vesicle glycoprotein 2A (SV2A) modulators such as ABBV-552 and AGB101, are in the phase I–III clinical trials for AD and for mild cognitive impairment (MCI) due to AD [14, 15].

Synaptic biomarkers may be used in biomarker panels for diagnostics, disease staging, and prediction of progression and have shown potential as a tool to monitor downstream effects on synaptic function and the integrity of treatments in drug trials [16–19]. Positron emission tomography (PET) using SV2A radioligands [^11^C]UCB-J, [^18^F]UCB-H, [^18^F]SynVesT-1, and [^18^F]SynVesT-2 [20–26] has been developed for in vivo imaging. SV2A is expressed at presynaptic terminals throughout the brain. It influences neurotransmitter release by regulating the amount of synaptotagmin (SYT1) in secretory vesicles, plays an important role in the trafficking of SYT1 to synaptic vesicles and regulates the effectiveness of calcium in inducing vesicle fusion. Synaptophysin (SYP) is an integral synaptic vesicle protein that modulates endocytosis and is crucial for trafficking the vesicular SNARE protein synaptobrevin II essential for neurotransmitter release [27, 28]. A reduction in SV2A protein level, which is related to tau deposition, has been demonstrated in the hippocampal and cortical regions of amnesic MCI patients with AD compared with nondemented controls (NCs) via in vivo imaging [29]. In addition, hippocampal SV2A tracer uptake in PET and serum levels of SV2A are positively correlated with cognitive performance in patients with AD and decrease with progression of AD [5, 30, 31]. In addition, fluid (cerebrospinal fluid [CSF] and blood) biomarkers based on ultrasensitive immunoassays, such as synaptosomal-associated protein 25 kDa (SNAP25), neurogranin, visinin-like protein 1, neuronal pentraxin 2, β-synuclein, SYT1, and growth-associated protein 43 (GAP43), have been identified [32–35]. Mass spectrometry (MS)-based proteomic analysis of CSF and plasma has demonstrated differentiation between sporadic AD patients and NCs as well as between sporadic AD and autosomal dominant AD patients [36–38]. In addition, emerging evidence has shown the potential of blood extracellular vesicles (EVs) carrying synaptic function- and brain-related proteins as potential biomarkers for AD [39].

The aims of this study were to (1) map the distribution and alterations of SV2A and SYP in the brains of AD patients compared with NCs; (2) assess the associations of SV2A and SYP with Aβ, tau, and the apolipoprotein E (*APOE*) ε4 allele in the brains of AD patients and NCs; and (3) understand the associations between SV2A and other synaptic markers in EVs. We included a total of 105 cases and performed MS-based proteomics on brain-derived EVs (BDEVs), and immunohistochemistry (IHC) and immunofluorescence (IF) staining of postmortem brain tissues.

## Materials and methods

### Postmortem human brain tissues

For the proteomics study, brain extracts from freshly frozen brain tissues from 17 patients with AD and 4 NCs were obtained from the Lille Neurobank and CHUV biobank, fulfilling requirements concerning biological resources, and were declared to the competent authority. The samples were managed by the CRB/CIC1403 Biobank (BB-0033–00030 for the Lille Neurobank and BB0066 for CHUV, Table 1). For the IHC/IF staining studies, postmortem paraffin-embedded brain tissues from 40 patients with AD, each with a clinical diagnosis confirmed by pathological examination, and 44 NCs were obtained from the Netherlands Brain Bank (NBB), Netherlands (Table 1), including the hippocampus, entorhinal cortex, frontal cortex and temporal cortex. We were not able to obtain sufficient numbers of freshly frozen hippocampal samples from AD and NCs to perform both proteomics analyses and IHC. Therefore, the tissues used for synaptic IHC and that used for proteomics came from different cases and different brain regions. All materials were donated with written informed consent for a brain autopsy and for the use of the materials and clinical information for research purposes from donors, obtained by the NBB, CHUV or Lille Neurobank. The study was conducted according to the principles of the Declaration of Helsinki and subsequent revisions. All the autopsied human brain tissue experiments were carried out in accordance with ethical permission obtained from the regional human ethics committee and the medical ethics committee of the VU Medical Center for NBB tissue. Information on *APOE* ε4 and the neuropathological diagnosis of AD (possible, probable, or definite AD) or not AD was obtained from the NBB. Information on the Consortium to Establish a Registry for AD (CERAD), which applies semiquantitative estimates of neuritic plaque density and the Braak stage [40] based on the presence of NFTs, was obtained from the NBB and CHUV and is provided in Table 1.

**Table 1:** Demographics and neuropathologic characteristics of brain tissue donors

	Proteomics cohort		IHC/IF cohort		Sum		
	NC (*n* = 4)	AD (*n* = 17)	NC (*n* = 44)	AD (*n* = 40)	NC (*n* = 48)	AD (*n* = 57)	Stat
Age, mean (SD), years	50.3 (11.8)	70.9 (2.7)	81.1 (10.4)	82.2 (7.7)	78.7 (12.7)	78.8 (9.4)	*t* = 0.0463; df = 103; *P* = 0.9632
Sex, *n* (%)							*χ*^2^ = 6.21; *P* = 0.0127
Female	1 (25)	9 (53)	25 (56.8)	35 (87.5)	26 (54.2)	44 (77.2)	
Male	3 (75)	8 (47)	19 (43.2)	5 (12.5)	22 (45.8)	13 (22.8)	
Braak stage							
0	3	0	6	0	9	0	
I	1	1	19	0	20	1	
II	0	2	8	3	8	5	
III–IV	0	4	10	10	10	14	
V–VI	0	10	0	25	0	35	
Amyloid CERAD							
O			18	0	18	0	
A			11	0	11	0	
B			7	4	7	4	
C			6	27	6	27	
PMD, mean (SD), h	15.0 (5.7)	20.8 (19.4)	7.1 (3.7)	4.3 (1.1)	7.8 (4.4)	9.2 (12.2)	*t* = 0.7543; df = 103; *P* = 0.4524
*APOE* ε4%	0	50.0	20.9	52.5	22.0	52.0	*χ*^2^ = 14.34 *P* = 0.0002
ε4 0 allele	0	5	34	19	34	24	
ε4 1 allele	0	1	9	13	9	14	
ε4 2 alleles	0	4	0	8	0	12	
Not defined	4	7	0	0	4	7	
SV2A; 4G8; AT8 IHC							
Hippo, EC	/	/	26	27	26	27	
PFC/FC	4*	17*	14	12	18	29	
TC	/	/	10	14	10	14	
SYP IHC							
Hippo, EC	/	/	24	25	24	25	
PFC/FC	/	/	0	5	0	5	
TC	/	/	0	4	0	4	

AD, Alzheimer’s disease; EC, entorhinal cortex; Hippo, hippocampus; TC, temporal cortex; FC, frontal cortex; PFC, prefrontal cortex; PMD, postmortem delay; SYP, synaptophysin. One AD patient did not have Braak stage information, and 9 AD patients did not have CERAD scores. *Postfixed frozen prefrontal cortex tissues was used for 4G8 and AT8 IHC for analysis of correlation with proteomics data. Fixed and paraffin-embedded frontal cortex tissues from the IHC/IF cohort were used for the staining of synaptic markers as well as 4G8 and AT8

### Interstitial fluid and BDEV isolation from patient prefrontal cortex tissues

Brain-derived fluids (BD-fluids) were isolated as previously described [41]. The prefrontal cortex was used for BDEV isolation because of the availability of high-quality, freshly frozen prefrontal cortex tissue and accumulation of amyloid and tau in the prefrontal cortex in AD patients. Briefly, approximately 1.5 g of freshly frozen prefrontal cortex was gently cut into smaller pieces, and then incubated in a Papain/Hibernate-E solution (Gibco, Thermo fisher, Waltham, MA) to gently digest the tissue and release interstitial fluid, theoretically avoiding cellular lysis. After papain digestion, ice-cold Hibernate-A medium supplemented with E64 (a specific cysteine protease inhibitor), a protease inhibitor cocktail (Roche, Basel, Switzerland), 50 mmol/L NaF, and 200 nmol/L Na₃VO₄ was added to ensure immediate and complete inhibition of enzymatic activity, including papain. Then the samples underwent differential centrifugation at 300 × *g* for 10 min, 2000 × *g* for 10 min, and 10,000 × *g* for 30 min at 4 °C to remove cells, membranes, and debris, respectively, and the final supernatant was stored at − 80 °C until EV isolation was performed. To avoid bias in the results, normalization according to the weight of the brain extracts was systematically performed. The procedures used to isolate BDEVs from human BD-fluid were carried out in accordance with the Minimal Information for the Studies of Extracellular Vesicles (MISEV) guidelines that were established and updated in 2024 by the International Society for Extracellular Vesicles [42]. Various controls were used to validate the enrichment and content of the BDEVs, as recommended in these guidelines. However, the procedure described above to recover BD-fluids may still lead to some cell lysis and therefore some intracellular contamination. The presence of intraluminal vesicles in the preparations cannot be fully excluded. A total of 500 μL of BD-fluid was loaded on top of a size exclusion chromatography column (10 mL column, pore size 75 nm, CL2B Sepharose, Merck-Millipore). A mean of 7.94 × 10^10^ (± 3.36 × 10^9^) vesicles/g of tissue in Faction 1–4 (F1-4) was recovered for each sample (*n* = 36 samples). Isolation was carried out in phosphate-buffered saline (PBS) with a flow of 36–48 s/mL. The first 3 mL was removed, and the following 20 fractions were recovered (with 500 μL per fraction).

### Label-free liquid chromatography tandem mass spectrometry (LC‒MS/MS)

F1‒4 fractions were digested according to a modified version of the intelligent Sample preparation Technology (miST) method [41, 43]. Briefly, 50 mL of solution in PBS was supplemented with 50 mL of miST lysis buffer (1% sodium deoxycholate, 100 mmol/L Tris pH 8.6, 10 mmol/L dithiothreitol) and heated at 95 °C for 5 min. Samples were then diluted 1:1 (*v*:*v*) with water, and reduced disulfides were alkylated by adding 1/4 volume of 160 mmol/L chloroacetamide (final 32 mmol/L) and incubating at 25 °C for 45 min in the dark. The samples were adjusted to 3 mmol/L ethylenediaminetetraacetic acid and digested with 0.5 mg of trypsin/LysC mixture (Promega #V5073, Madison, WI) for 1 h at 37 °C, followed by a second 1 h of digestion with a second and identical aliquot of proteases. To remove sodium deoxycholate and desalted peptides, two sample volumes of isopropanol containing 1% trifluoroacetic acid were added to the digests, and the samples were desalted on a strong cation exchange plate (Oasis MCX; Waters Corp., Milford, MA) by centrifugation. After washing with isopropanol/1% trifluoroacetic acid, the peptides were eluted in 250 mL of 80% acetonitrile, 19% water, and 1% (*v*/*v*) ammonia. Tryptic ptide mixtures were then injected on an Ultimate RSLC 3000 nanoHPLC system (Thermo Fisher, Waltham, MA) interfaced via a nanospray Flex source to a high-resolution Orbitrap Exploris 480 mass spectrometer (Thermo Fisher, Bremen, Germany). Peptides were loaded onto an Acclaim PepMapocolumn (20 mm × 100 μm ID, 5 μm, Dionex) before separation on a C18 custom-packed column (75 μm ID × 45 cm, 1.8 μm particles, Reprosil Pur, Dr. Maisch), and a gradient from 4% to 90% acetonitrile in 0.1% formic acid was used for peptide separation (total time: 140 min). Full MS survey scans were performed at 120,000 resolution. A data-dependent acquisition method controlled by Xcalibur software (Thermo Fisher Scientific) was used to optimize the number of precursors selected ("top speed") to charge 2 + to 5 + while maintaining a fixed scan cycle of 2 s. Peptides were fragmented by higher energy collision dissociation with a normalized energy of 30% at 15,000 resolution. The window for precursor isolation was 1.6 m/z units around the precursor, and selected fragments were excluded for 60 s from further analysis.

### MS and MS data analysis

The data files were analyzed with MaxQuant 1.6.14.0 (Max-Planck-Institut für Biochemie, Martinsried**,** Germany), which incorporates the Andromeda search engine [44]. Cysteine carbamidomethylation was selected as a fixed modification, whereas methionine oxidation and protein N-terminal acetylation were specified as variable modifications. The sequence databases used for searching were the human (*Homo sapiens*) reference proteome from the UniProt database and a "contaminant" database containing the most common environmental contaminants and enzymes used for digestion (keratins, trypsin, etc.). The mass tolerance was 4.5 ppm for the precursors (after recalibration) and 20 ppm for the MS/MS fragments. Both peptide and protein identifications were filtered at a 1% false discovery rate relative to hits against a decoy database built by reversing protein sequences. Proteins were annotated according to several databases. These databases include the Gene Ontology (GO) Human database, which is further split into the categories of cellular component, biological process, and molecular functions, as well as the MISEV guidelines for EV-associated and potential contaminant proteins, a database generated by McKenzie and colleagues in their publication for brain cell type specificity, and the Human Protein Atlas databases for cell type specificity and predicted subcellular localization. Within the neuronal category, we also compared excitatory and inhibitory neuron-specific proteins according to the Human Protein Atlas [45].

### Weighted gene coexpression network analysis (WGCNA)

Protein coexpression network analysis was performed with the R package WGCNA [46] on the preprocessed proteomic data of all identified proteins with their respective riBAQ scores for each disease group. First, a correlation matrix for all pairwise correlations of proteins across all samples was generated and then transformed into a matrix of connection strengths, i.e., a weighted adjacency matrix with soft threshold power β = 16. The topological overlap (TO) was subsequently calculated via the connection strengths. Proteins were further hierarchically clustered via the 1-TO measure as the distance measure to generate a cluster dendrogram, and modules of proteins with similar coexpression relationships were identified via the dynamic tree-cutting algorithm with the following parameters: minimal module size = 30, deepSplit = 2, and merge cut height = 0.15. Within each module, the module eigenprotein was defined as the first principal component, which serves as a weighted summary of protein expression within the module and accounts for the greatest variance among all module proteins. Furthermore, module membership (kME) was assigned by calculating Pearson’s correlation between each protein and each module eigenprotein and the corresponding* P* value. Proteins were reassigned to the module for which they had the highest module membership, with a reassignment threshold of *P* < 0.05. From each module, hub proteins were identified via the signedkME function, which explains the membership of a protein with its module and its strong associations with other proteins within the module.

### Gene ontology

A detailed genetic annotation of each protein within the WGCNA modules was performed. These differentially expressed proteins and coexpressed proteins were characterized on the basis of their gene ontologies via the GO-Elite (version 1.2.5) python package [47]. The entire set of proteins identified and included in the network analysis served as the background dataset. The presence of significantly overrepresented ontologies within a module was gauged via a Z score, whereas the significance of the Z scores was evaluated via a one-tailed Fisher’s exact test, with adjustments for multiple comparisons via the Benjamini‒Hochberg false discovery rate method. The threshold analysis included a Z score cutoff of 2, a* P* value threshold of 0.05, and a minimum requirement of five genes per ontology before ontology pruning was performed. The best gene ontology term, which explains the molecular and cellular functions of each module, was used for naming.

### Protein‒protein interaction network

Proteins used as inputs for network generation were selected from significant WGCNA modules. The Signaling Network Open Resource (SIGNOR) database (ref SIGNOR app + specific tool used) was used to generate an interaction network in Cytoscape (v.3.10) for each significant module [48]. The obtained networks were merged into a single network that was curated to keep only proteins present in our data and the most prominent subnetworks. Finally, information on module appartenance and correlation with SV2A was added to improve the visual representation and relevance of the network.

### IHC, IF staining, microscopy and image analysis

The frozen brain tissues (from 17 AD patients and 4 NCs) adjacent to the tissues used for proteome analysis were cryosectioned to 20-μm sections, fixed and used for IHC staining to assess pathology using Aβ_17-24_ (4G8) and p-tau (AT8, Ser202/Thr205) antibodies and its association with BDEV markers. Nissl and Gallyas staining were also performed on frontal cortex tissue slices (20-μm section) after fixation. SV2A and SYP were stained on slices from paraffin-embedded fixed postmortem brain tissues, as frozen tissues may lead to freezing artifacts for quantification of synaptic markers.

The paraffin-embedded fixed postmortem brain tissues (from 40 AD patients and 44 NCs) were cut into 3-µm sections via a Leica microtome (Leica Microsystems, Germany). Hematoxylin and eosin (H&E) staining was performed for each patient according to routine procedures to provide anatomical information and to determine whether there were abnormalities in the brain. IHC staining with antibodies against SV2A, SYP, and Aβ_17-24_ (4G8) was performed in the hippocampus, entorhinal cortex, frontal cortex and temporal cortex [49, 50]. IF staining using antibodies against phospho-tau (AT8, Ser202/Thr205) was performed in the hippocampus and entorhinal cortex. To assess the spatial associations of SV2A with Aβ and tau, double IF staining was performed using SV2A/6E10 (antibodies against Aβ_1-16_) and SV2A/AT8 on the frontal cortex, temporal cortex and hippocampus/entorhinal cortex slices from AD patients and NCs. The details of the antibodies, kits and chemicals used are listed in Table S1. Paraffin-embedded fixed human brain tissue sections were permeabilized and blocked in 5% normal donkey or goat serum and 1% Triton-phosphor-buffered saline (PBS) for one hour at room temperature with mild shaking. The slices were then incubated with primary antibodies overnight at 4 °C with mild shaking. The next day, the sections were washed with PBS two times for 20 min and incubated with secondary antibody for 2 h at room temperature. The sections were incubated for 15 min in 4′,6-diamidino-2-phenylindole, washed two times for 10 min with 1 × PBS, and mounted with VECTASHIELD Vibrance Antifade Mounting Media (Vector Laboratories, Newark, CA). The brain sections were imaged at × 20 magnification via an Axio Oberver Z1 slide scanner (Zeiss, Jena, Germany) and × 20 magnification via Leica SP8 confocal microscopy (Leica, Wetzlar, Germany) via the same acquisition settings.

For the analysis of staining results in the PFA-fixed tissues, manual delineation of hippocampal cornu ammonis (CA) 1, CA 2/3, dentate gyrus (DG), subiculum, entorhinal cortex, gray matter and white matter in the frontal and temporal cortex was performed using the Allen atlas as a reference [51]. For the analysis of staining results in the postfixed frozen tissues, no segmentation of white matter or gray matter was conducted to provide a more comprehensive reflection of the amyloid and tau levels across the entire tissue, which is more relevant to the proteomics data. Qupath (open source software) [52] and ImageJ (NIH, Bethesda, MD) were used for image processing and analysis. The percentage of positively stained area was quantified using the MaxEntropy autothreshold plugin in ImageJ for IHC staining, and the mean fluorescence intensity was quantified for IF staining.

### Statistics

All statistical analyses were performed with GraphPad Prism 10 (GraphPad Software, Boston, MA). The Shapiro‒Wilk test was used to assess the normality of distributions of various parameters in both groups. For analysis of difference of numerical variables between two groups, the nonparametric Mann‒Whitney U test was used. For analysis of differences in the numerical variables among multiple groups, one-way ANOVA with Tukey’s post hoc test was used. The chi-square test was used to assess differences in categorical variables, such as sex and *APOE* ε4 status between two groups. Nonparametric Spearman’s rank correlation analysis was performed for those that failed the normality test. Significance was set at *P* < 0.05. Data are shown as mean ± SD.

## Results

### Demographics and pathological description

Demographics of the donors included in this study are presented in Table 1. The ages in the NC group (78.7 ± 12.7 years, *n* = 48) were comparable to those in the AD group (78.8 ± 9.4 years, *n* = 57) and followed a normal statistical distribution (Shapiro‒Wilk test). In the IHC/IF cohort, the age was comparable between the NC (81.1 ± 10.4 years, *n* = 44) and AD groups (82.2 ± 7.7 years, *n* = 40, *P* = 0.77). In the proteomics cohort, the age was lower in the NC group (50.3 ± 11.8 years, *n* = 4) compared with the AD group (70.9 ± 2.7 years, *n* = 17, *P* = 0.07), but with no significant difference. The ages of the NC and AD groups in the proteomics analysis were lower than those in the IHC/IF analysis (*P* < 0.0001, *P* < 0.0001). The postmortem delay (PMD) was longer in the NC group (7.1 ± 3.7 h) than in the AD group (4.3 ± 1.1 h) for IHC/IF cohort (*P* < 0.0001). The PMD of brain tissues was comparable between the NC (15.0 ± 5.7 h) and AD groups (20.8 ± 19.4 h, *P* = 0.09) in the proteomics cohort. The PMDs in the proteomics cohort were greater than those in the IHC/IF cohort for both NC (*P* = 0.014) and AD (*P* < 0.0001). Our analysis revealed that the PMD in sample collection did not significantly influence the EV yield or protein content; however, this factor remains important to consider when working with postmortem samples (Fig. S1).

A greater percentage of females was noted in the AD group (77.2%) than in the NC group (54.2%) (*χ*^2^ = 6.21, *P* = 0.0127). The prevalence of *APOE* ε4 carriers was greater in the AD group (52%) than in the NC group (22%) (*χ*^2^ = 14.34, *P* = 0.0002). There were no NC carrying two *APOE* ε4 in the current samples. H&E staining revealed no abnormalities in the brain tissue slices from the AD patients or NCs (examples shown in Fig. S2). The specific information on age, sex, PMD, and *APOE* ε4 distribution of the cases included for proteomics analysis and for IHC/IF staining are shown in Table 1.

We used 4G8 IHC staining to map Aβ distribution in the hippocampus, entorhinal cortex, frontal cortex and temporal cortex slices and AT8 IF staining to map pathological phospho-tau distribution in the hippocampus and entorhinal cortex slices (thickness 3 μm) (Fig. S3, Fig. S4). Aβ plaques and tau pathology were also observed in the brains of a proportion of NCs (Table 1). For the frozen tissues (adjacent to the tissues used in proteomics), 4G8 and AT8 staining were performed after fixation of the tissue (thickness 20 μm).

### SV2A expression in BDEVs was lower in the AD brains than in the NC brains and emerged as a central hub in the ‘synaptic vesicle’ module related to synaptic vesicle formation and fusion

**Fig. 1:**
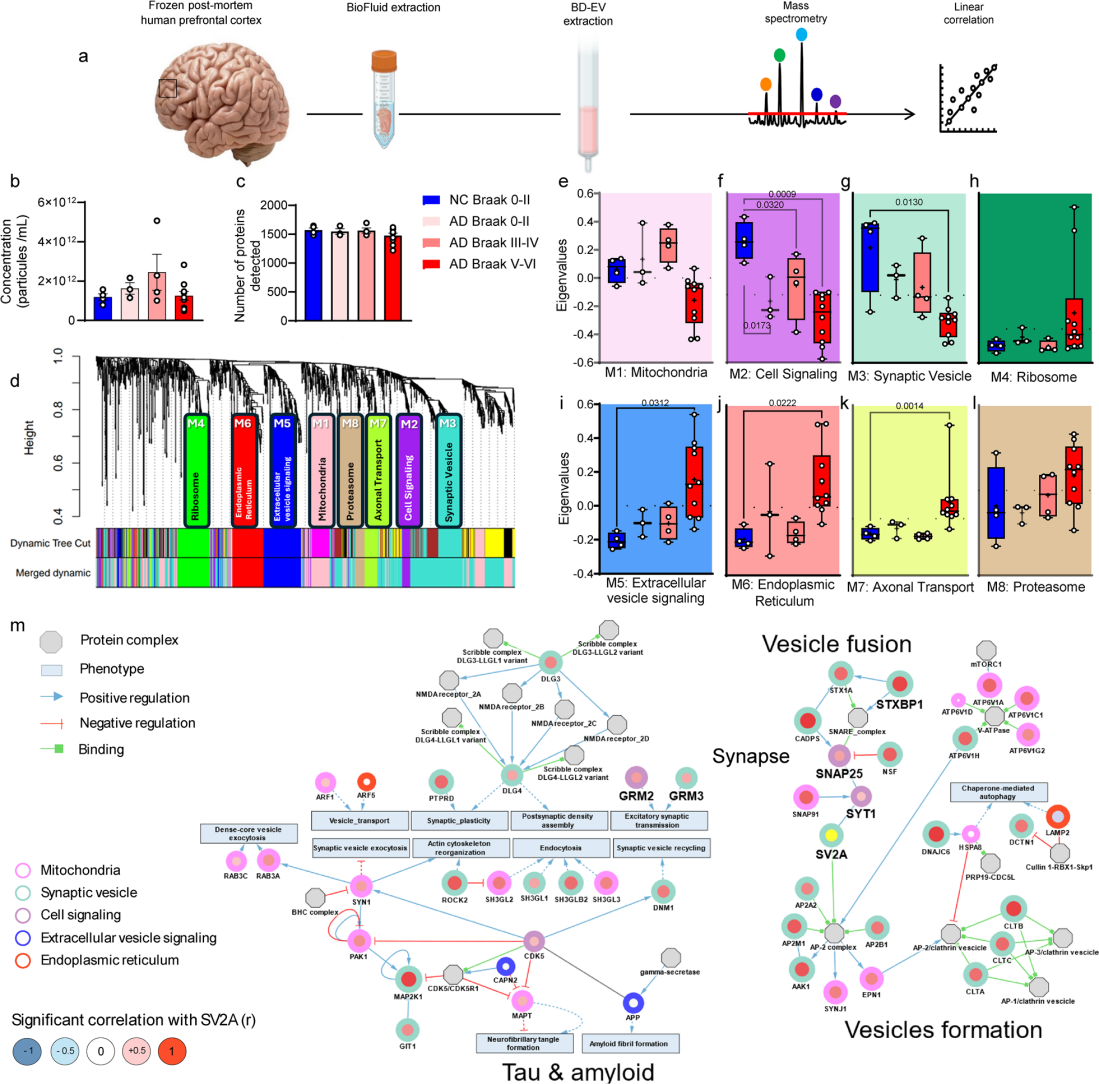
Proteomics of BDEVs from AD and NC brains. **a** Schemetic of experimental flow for brain-derived EV proteomics. **b** Histogram showing the relative concentrations of particles in the NCs and ADs of different Braak stages. **c** Histogram showing the relative quantities (rIBAQs) of proteins in the NCs and ADs of different Braak stages. **d** WGCNA cluster dendrogram and significant modules. **e**–**l** Comparisons of module eigenvalues between NCs and ADs of different Braak stages for mitochondria (**e**), cell signaling (**f**), synaptic vesicles (**g**), ribosomes (**h**), extracellular vesicle signaling (**i**), the endoplasmic reticulum (**j**), axonal transport (**k**), and proteasomes (**l**). **m** Protein‒protein interaction networks for proteins of the WGCNA modules (circles), with additional protein complexes (octagons) and phenotypes (rectangles). Border color indicates the module of the protein, and fill color indicates the *r* of correlative analysis (Pearson’s correlation; red = positive, *r* > 0.5; blue = negative, *r* < 0.5). Lines with different colors indicates different types of interaction (black: unspecified; blue: positive regulation; red: negative regulation; and green: binding). The dashed lines indicate indirect interactions

Following a previously published protocol [41], we isolated BDEVs from brain biofluids collected from the prefrontal cortex of 4 NCs and 17 patients with AD at different stages. We employed a previously published method, combining slow digestion of freshly frozen patient tissues with size-exclusion chromatography [41]. A portion of the isolated vesicles was analyzed via nanoparticle tracking analysis to quantify their concentration and size, whereas another fraction was digested with trypsin for in-depth analysis via LC‒MS/MS (Fig. 1a). Our results revealed that neither the number of vesicles (Fig. 1b) nor the number of proteins detected (Fig. 1c) varied significantly between the NC and AD groups, regardless of disease stage. Annotation of the identified proteins according to the MISEV2023 guidelines further confirmed that the relative abundance of EV-associated proteins, as well as potential contaminants, remained stable across groups (Fig. S5a–c). These initial quality control analyses indicate that the progression of AD does not impact the quality of the BDEVs isolated from the prefrontal cortex of AD patients. Nevertheless, we observed a significantly greater quantity of proteins CD9 and HSPG2 in the BDEVs of AD Braak V–VI patients, suggesting that some EVs could be enriched with CD9, which could reflect a redistribution of certain proteins at the late Braak stage (Fig. S5d–h). Next, we further assessed the relative abundance of brain-cell type-specific proteins within the EV proteome of NCs and AD patients at different Braak stages (Fig. S6). We observed significantly lower neuronal protein levels in the EVs of AD Braak 0–II and AD Braak V–VI compared to NCs (*P* = 0.0109, *P* < 0.0001) and significantly higher neuronal protein levels in the EVs of AD Braak 0–II and Braak III–IV compared to AD Braak V–VI patients (*P* = 0.0026, *P* < 0.0001). In contrast, there was a significantly higher level of oligodendrocyte-derived proteins in the EVs of AD Braak 0–II and Braak V–VI groups compared to NCs (*P* = 0.0486, *P* = 0.0050, respectively).

To investigate the global proteomic signature of BDEVs, we applied a correlation-based approach involving WGCNA, which identified eight functional modules (Fig. 1d–l). Among these modules, the M2 ("Cell signaling") (Fig. 1f) and M3 ("Synaptic vesicles") (Fig. 1g) modules were significantly reduced in AD patients. In contrast, the M6 ("endoplasmic reticulum") (Fig. 1j) and M7 ("axonal transport") (Fig. 1k) modules significantly increased at advanced disease stages (Braak V–VI). The M3 module was the only module significantly altered from early stage and across all stages, suggesting a strong association between BDEV proteomic signatures and AD progression, particularly concerning synaptic proteins.

**Fig. 2:**
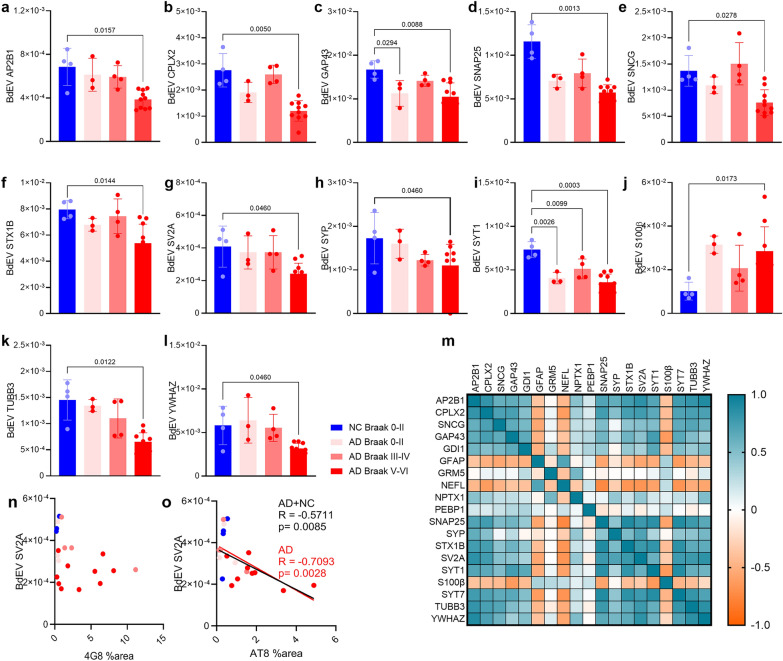
Comparison of cortical BDEV markers between NC and AD brains and correlation analysis. **a**–**l** BDEV levels of AP2B1, CPLX2, GAP43, SNAP25, SNCG, STX1B, SV2A, SYP, SYT1, S100β, TUBB3 and YWHAZ in the NC Braak 0–II, and AD of Braak 0–II, III–IV, and V–VI. **m** Nonparametric Spearman’s rank analysis of the rIBAQ matrix of correlations in the AD + NC pooled group (detailed *P* values and *r* values are provided in Table 2 and supplemental files). **n**, **o** Nonparametric Spearman’s rank analysis revealed a negative correlation between BDEV SV2A and AT8 tau but not with 4G8 Aβ % area in the prefrontal cortex tissue block (including gray and white matter)

To explore the modules further, we analyzed protein interactions via the SIGNOR platform (Fig. 1m). Two main subnetworks were identified, highlighting proteins involved in synaptic functions, vesicular transport, and proteopathic mechanisms related to tau and Aβ. Within the M3 module, the protein SV2A emerged as a central hub due to its numerous interactions with proteins from subnetworks related to synaptic vesicle formation and fusion. To better understand the role of SV2A in BDEVs, we conducted linear correlation analyses between its relative abundance and that of other proteins in the modules. We found that many proteins within associated clusters were positively correlated with SV2A (nodes with red color in the center). These proteins were predominantly located within the "Synaptic vesicles", "Mitochondria", and "Cell signaling" modules (Fig. 1m). Finally, to assess whether SV2A and other synaptic markers are reduced in BDEVs and whether SV2A levels are correlated with other recently proposed CSF panel biomarkers of synaptic dysfunction in neurodegenerative diseases [53], we performed nonparametric Spearman’s rank correlation analyses. Among this panel of 25 proteins, 21 were detectable in BDEVs (Table 2). Notably, we observed significant decreases in proteins at the late stage of AD (AD Braak stages V–VI), including adaptor-related protein complex 2 subunit beta 1 (AP2B1), complexin-2 (CPLX2), GAP43, SNAP25, gamma-synuclein, SV2A, syntaxin 1B, SYP, SYT1, tubulin beta 3 class III (TUBB3) and 14-3-3ζ (Fig. 2a–l). Interestingly, only GAP43 and SYT1 were significantly reduced in BDEVs from early stage of the disease (AD Braak stages 0-II) (Fig. 2c, i). In contrast, no differences in the levels of GDP dissociation inhibitor 1, neuronal pentraxin 1, neurofilament light chain (NEFL), phosphatidylethanolamine binding protein 1, SYT7 or postsynaptic metabotropic glutamate receptor subtype 5 were detected between AD patients and NCs (Fig. S7). Compared with the NC group, the level of the neuroinflammation-associated marker S100 calcium-binding protein β, but not glial fibrillary acidic protein (GFAP), was increased in the Braak stage V–VI AD group (Fig. 2j, Fig. S7).

**Table 2:** Nonparametric spearman rank correlation analysis between SV2A, SYP and other markers in the BDEVs of AD and NC brains

Proteins	Detectable	SV2A				SYP			
		AD + NC		AD		AD + NC		AD	
		*R*	*P* value	*R*	*P* value	*R*	*P* value	*R*	*P* value
AP2B1	Yes	0.8460	** < 0.0001**	0.8460	** < 0.0001**	0.6273	**0.0023**	NS	NS
CHAT	No								
CHRNA7	No								
CMPLX2	Yes	0.7609	**0.0001**	0.7609	**0.0001**	0.4857	**0.0256**	NS	NS
GAP43	Yes	0.4587	**0.0365**	0.4587	**0.0365**	NS	NS	NS	NS
GDI-1	Yes	0.3502	0.1196	0.3502	0.1196	NS	NS	NS	NS
GFAP	Yes	−0.5608	**0.0082**	−0.5608	**0.0082**	NS	NS	NS	NS
GRM5	Yes	−0.0023	0.9921	−0.0023	0.9921	NS	NS	NS	NS
NEFL	Yes	−0.6725	**0.0008**	−0.6725	**0.0008**	NS	NS	NS	NS
NPTX1	Yes	0.4228	0.0562	0.4228	0.0562	NS	NS	NS	NS
NPTX2	Yes	NS	NS	NS	NS	NS	NS	NS	NS
NPTXR	Yes	NS	NS	NS	NS	NS	NS	NS	NS
NRGN	No								
PEBP-1	Yes	−0.0981	0.6722	−0.0981	0.6722	NS	NS	NS	NS
SNAP25	Yes	0.7843	** < 0.0001**	0.7843	** < 0.0001**	0.4494	**0.0409**	NS	NS
SNCB	No								
SNCG	Yes	0.4873	**0.0250**	0.4873	**0.0250**	NS	NS	NS	NS
STX1B	Yes	0.7888	** < 0.0001**	0.7888	** < 0.0001**	0.5247	**0.0146**	NS	NS
SV2A	Yes	/	**/**	/	**/**	0.7063	**0.0003**	0.5236	**0.0328**
SYP	Yes	0.7063	**0.0003**	0.5236	**0.0328**	NS	NS	NS	NS
SYT-1	Yes	0.6023	**0.0039**	0.6023	**0.0039**	NS	NS	NS	NS
SYT-7	Yes	0.7781	** < 0.0001**	0.7781	** < 0.0001**	0.4434	**0.0441**	NS	NS
S100β	Yes	NS	NS	NS	NS	NS	NS	NS	NS
TUBB3	Yes	0.7797	** < 0.0001**	0.7797	** < 0.0001**	0.4532	**0.0391**	NS	NS
VILIP-1	No							NS	NS
14-3-3ζ	Yes	0.8369	** < 0.0001**	0.8369	** < 0.0001**	0.5519	**0.0095**	NS	NS

NS, not significant; Significance [bold]: *P* < 0.05; AP2B1, adaptor-related protein complex 2 subunit beta 1; CHAT, choline acetyltransferase; CHRNA7, α7 nicotinic acetylcholine receptor; CPLX2, complexin-2; GAP43, growth-associated protein 43; GDI-1, guanosine diphosphate (GDP) dissociation inhibitor-1; GFAP, glial fibrillary acidic protein; GRM5, metabotropic glutamate receptor subtype 5; NPTX1, neuronal pentraxin 1; NEFL, neurofilament light chain; PEBP1, phosphatidylethanolamine binding protein 1; S100β, S100 calcium-binding protein β; SNAP25, synaptosomal-associated protein 25 kDa; SNCG, gamma-synuclein; SV2A, synaptic vesicle glycoprotein 2A; STX1B, syntaxin 1B; SYP, synaptophysin; SYT1, synaptotagmin 1; SYT7, synaptotagmin 7; TUBB3, tubulin beta 3 class III

### SV2A is positively correlated with other synaptic proteins and negatively correlated with Aβ and p-tau levels

Next, we analyzed the correlation of different BDEV markers in the AD + NC group (Fig. S8) and the AD group (Fig. S9). Among these 24 synaptic proteins, 12 were positively correlated with the relative levels of SV2A, with AP2B1, 14-3-3ζ, SNAP25, syntaxin 1B, SYT7, TUBB3 and CPLX2 showing the highest correlations (*r* > 0.75, *P* < 0.0001) (Fig. 2, Table 2, Fig. S10), indicating synaptic and neuronal loss. In comparison, the correlation between the relative levels of SYP and the above 7 synaptic proteins was weaker (all *r* values < 0.75). The negative correlation between GFAP and synaptic proteins indicates concomitant astrogliosis with synaptic dysfunction, with the strongest correlations with SYT7 and SV2A (*r* > 0.75, *P* < 0.0001) (Fig. 2, Table 2). Moreover, to understand the association of SV2A with phospho-tau and Aβ, correlation analysis was performed between BDEV SV2A and the IHC measurements of the phospho-tau AT8% area and Aβ 4G8% area of the adjacent tissue slices. Negative correlations were detected in the AD + NC group between SV2A level and the tau AT8% area (*P* = 0.0085, *r* = − 0.5711) and in the AD group between SV2A level and the tau AT8% area (*P* = 0.0028, *r* = − 0.7093). No correlation was detected in the AD + NC group or in the AD group between SV2A and the Aβ 4G8% area. These data suggest that the synaptic markers in BDEVs are particularly central in detecting the progression of the pathology and that SV2A seems to be a central protein that correlates well with other proteins. Therefore, SV2A may serve as a valuable marker for synaptic degeneration in AD.

### Reduced levels of SV2A in AD patients compared with NCs and in *APOE* ε4 allele carriers compared with noncarriers

**Fig. 3:**
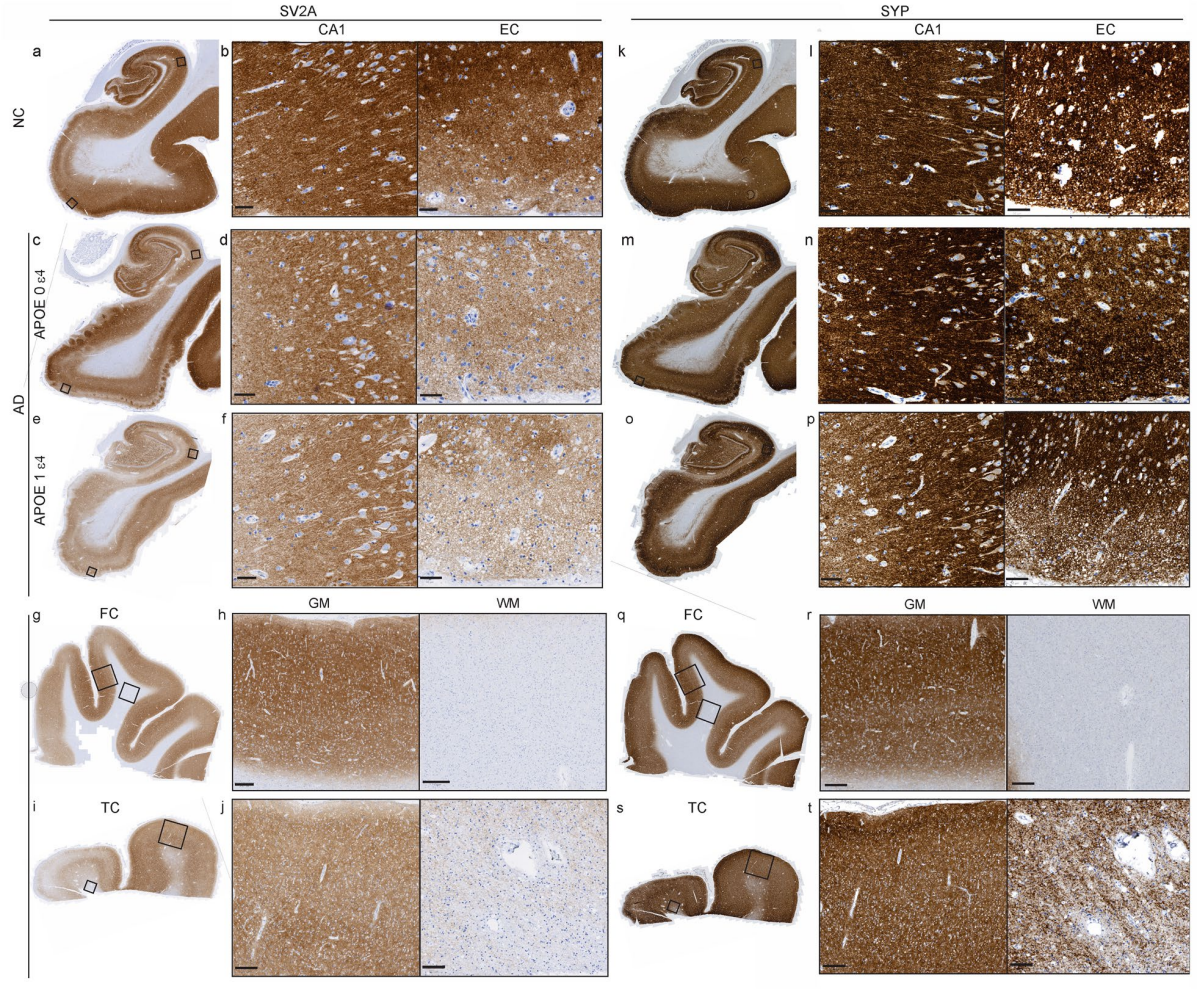
SV2A and SYP staining in the hippocampus, entorhinal cortex, frontal cortex and temporal cortex of NC and AD *APOE* ε4 carriers and noncarriers. **a**–**f**, **k**–**p** Representative images of IHC staining for SV2A (**a**–**f**) and SYP (**k**–**p**) in the hippocampi of the NC and AD *APOE* ε4 noncarriers and carriers. Zoomed-in images of the CA1 region and the entorhinal cortex (EC). **g**–**j**, **q**–**t** Representative images of IHC staining for SV2A (**g**–**j**) and SYP (**q**–**t**) in the frontal cortex (FC) and temporal cortex (TC) of AD patients. Zoom-in images of gray matter (GM) and white matter (WM) in the cortex regions. Scale bars, 50 μm (**b**, **d**, **f**, **l**, **n**, **p**), 100 μm (**j**, **t**), and 400 μm (**h**, **r**)

**Fig. 4:**
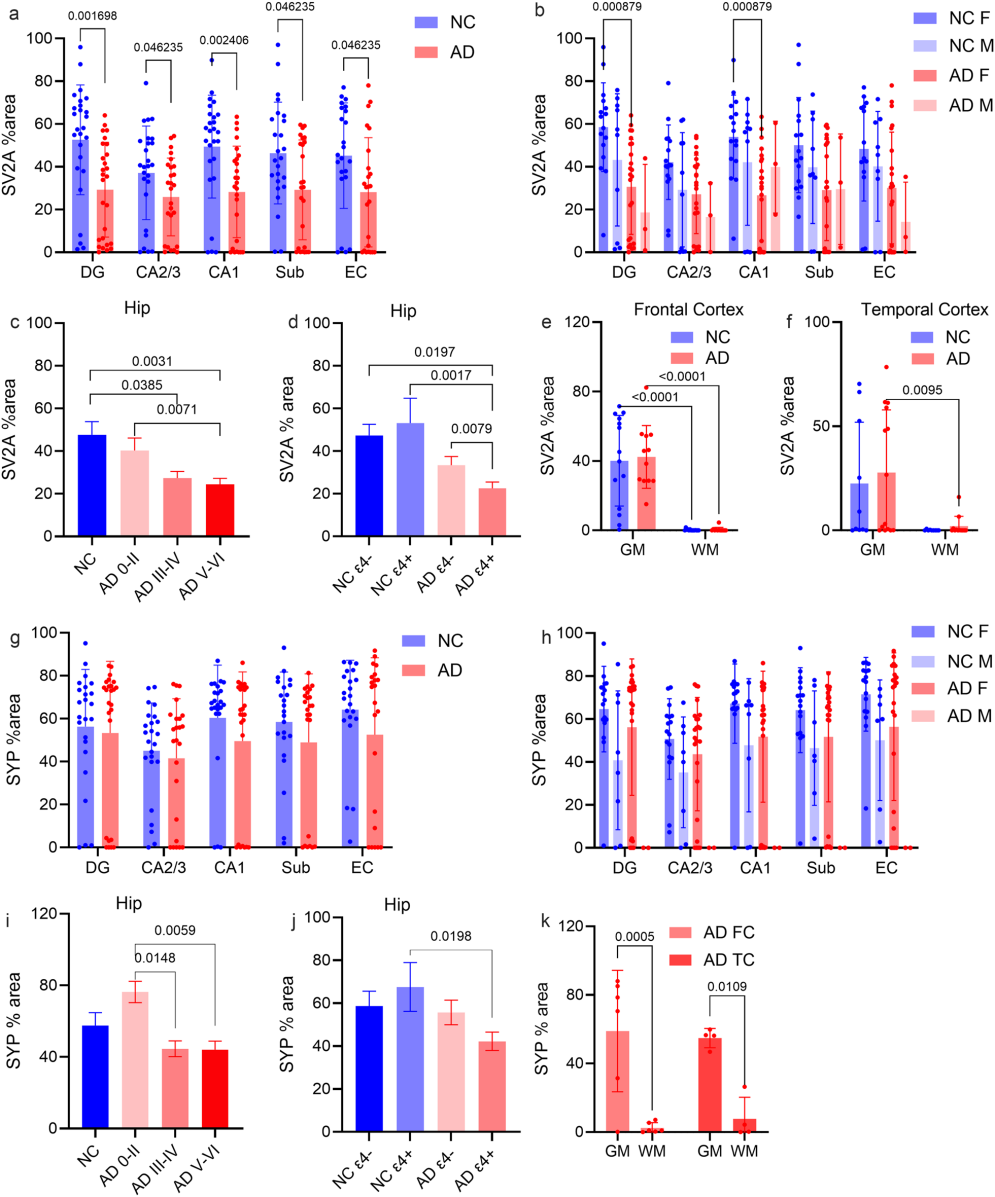
Reduced SV2A but not SYP levels in the hippocampus and entorhinal cortex of AD patients compared with NCs and associations with the *APOE* ε4 and Braak stage. **a**, **b**, **g**, **h** SV2A and SYP levels in the dentate gyrus (DG), CA3/CA2, CA1, and subiculum (Sub) of the hippocampus and the entorhinal cortex (EC) in AD patients compared with those in NCs (SV2A: NC *n* = 26, AD *n* = 27; SYP: NC *n* = 24, AD *n* = 25) (**a**, **g**) and in female and male AD patients and NCs (**b**, **h**). **c**, **i** Comparison of hippocampal SV2A and SYP levels between NC and AD patients at different Braak stages. **d**, **j** SV2A and SYP levels in *APOE* ε4 carriers and noncarriers of AD and NCs. **e**, **f**, **k** SV2A and SYP levels were higher in the gray matter (GM) than in the white matter (WM) of the frontal cortex (FC) or temporal cortex (TC) (SV2A FC, NC *n *= 14, AD *n* = 12; SV2A TC, NC *n* = 10, AD *n* = 14; SYP FC, AD *n* = 5; SYP TC, AD *n* = 4)

We attempted IHC for synaptic markers using postfixed frozen tissues from our available prefrontal cortex samples; however, the staining quality was not satisfactory. As a result, the tissues used for synaptic IHC and for proteomics were from different cases. We first quantified SV2A protein levels in brain tissue slices from the hippocampus, entorhinal cortex, frontal cortex, and temporal cortex of AD patients and NCs by IHC analysis. The SV2A level did not differ significantly among subfield regions of the hippocampus. Compared with the NCs, SV2A levels were lower in all the hippocampal subfields of AD patients, namely, the DG (*P* = 0.0017), CA2/3 (*P* = 0.0462), CA1 (*P* = 0.0024), and subiculum (*P* = 0.0462), as well as in the entorhinal cortex (*P* = 0.0462) (Figs. 3a–f and 4a, Fig. S11). In the frontal cortex and temporal cortex of both AD patients and HCs, the level of SV2A in the gray matter was several-fold greater than that in the white matter (Figs. 3g–j and 4e, f). The levels of SV2A in the gray matter or white matter in the frontal cortex and temporal cortex of AD patients were comparable to those in NCs based on IHC staining (Figs. 3 and 4e, f, Fig. S11). The laminar distribution of SV2A in the gray matter of the frontal, temporal and entorhinal cortex is rather homologous. In contrast, the pyramidal layer of the hippocampus presented a dense SV2A signal.

The SYP level did not differ significantly between AD patients and NCs in the subfields of the hippocampus or the entorhinal cortex (Figs. 3k–p and 4g–j). Similar to SV2A*,* in the frontal cortex and temporal cortex of both AD patients and NCs, the level of SYP in gray matter was several-fold greater than that in white matter (Figs. 3q–t and 4k). Like SV2A, the laminar distribution of SYP in the gray matter of the frontal, temporal and entorhinal cortex is mostly homogenous; the pyramidal layer of the hippocampus shows dense SYP signals (Fig. 3).

Next, we assessed the effects of the *APOE* ε4 allele and sex on SV2A and SYP levels. We found that SV2A levels were lower in AD *APOE* ε4 carriers than in AD noncarriers (Figs. 3c–f and 4d, *P* = 0.0079) but this effect was not found for SYP level (Figs. 3m–p, 4j). A lower level of SYP was observed in AD *APOE* ε4 carriers than in NC *APOE* ε4 carriers (Fig. 4j). There was no significant difference in the level of SV2A or SYP between female and male AD patients or between female and male NCs (Fig. 4b, h).

### Regional levels of SV2A are positively correlated with SYP and negatively correlated with Aβ, phospho-tau, and the Braak stage

Higher levels of SV2A (Fig. 4c, *P* = 0.0071) were detected in AD at Braak stages 0–II than in V–VI, similar as the higher levels of SYP in AD at Braak stages 0–II than at stages V–VI (Fig. 4i, *P* = 0.0059) and III–IV (Fig. 4i, *P* = 0.0148). Compared with NCs, SV2A levels were lower in the AD patients at Braak stages III–IV, as well as at Braak stages V–VI (Fig. 4c, *P* = 0.0385, *P* = 0.0031), but not in the AD patients at Braak stages 0–II. In contrast, no difference in the levels of SYP was detected between the NC and AD groups at different Braak stages (Fig. 4i).

**Fig. 5:**
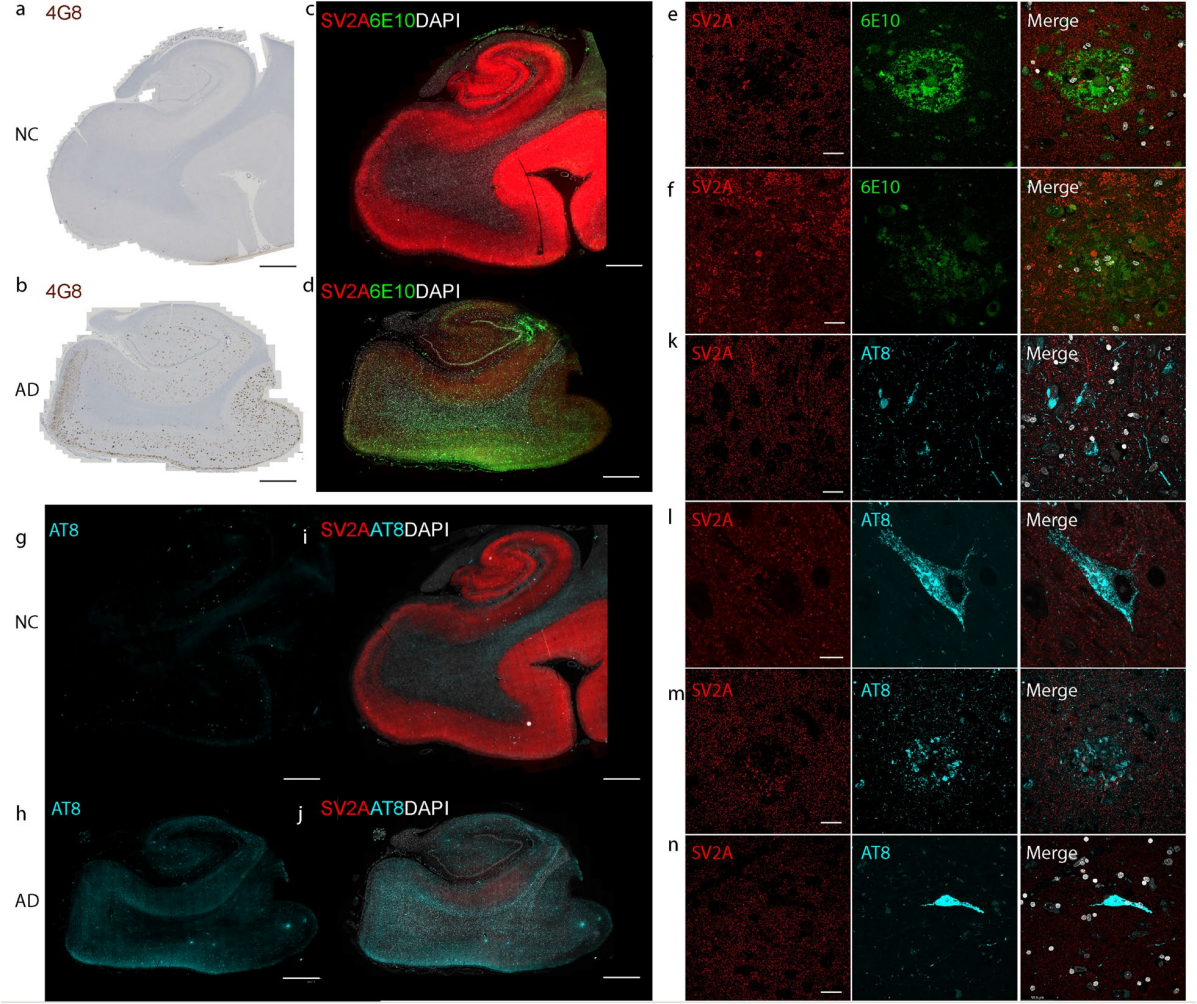
SV2A protein level is lower in the presence of amyloid-β plaques (4G8, 6E10) and phospho-tau (AT8) in AD patients than in NCs. **a**, **b** Representative overview of 4G8 amyloid-β (brown) IHC staining in the NC and AD groups. **c**–**n** Representative overview and zoomed-in view of immunofluorescence staining of SV2A (red), 6E10 amyloid-β (green) and AT8 (cyan) in the hippocampi of the NC and AD groups. Nuclei were counterstained with DAPI (white). Core plaque (**e**), diffuse plaque (**f**), neuropil thread (**k**), mature tangle (**l**), neuritic plaque (**m**), and ghost tangle (**n**) in AD brain. Scale bars, 2 mm (**a**–**d**, **g**–**j**) and 20 μm (**e**, **f**, **k**–**n**). DAPI: 4′,6-diamidino-2-phenylindole

**Fig. 6:**
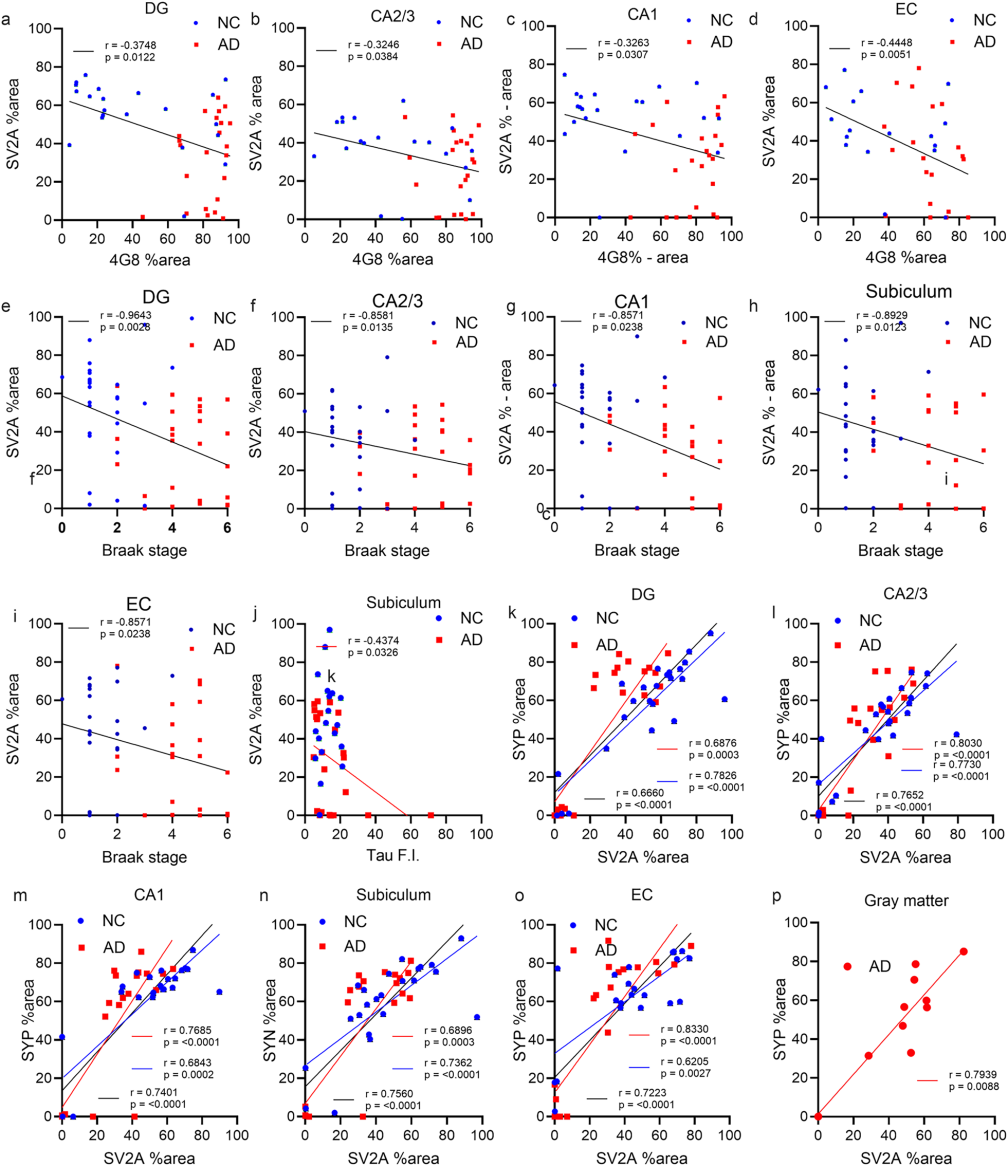
Correlation analysis for SV2A and SYP levels with amyloid-β, Braak stage and tau. **a**–**d** Negative correlations between 4G8 amyloid-β and SV2A levels in the hippocampus and EC in the AD + NC pooled group. **e**–**i** Negative correlations of SV2A levels with Braak stage in the hippocampus and EC regions in the AD + NC pooled group. **j** SV2A and AT8 (phospho-tau) levels were negatively correlated in the subiculum of AD patients. **k**–**p** Positive correlations between SYP and SV2A levels in different regions in AD patients and NCs. AD; red line, NC; black line, AD + NC. Detailed *P* and *r* values are provided in Table S2

Next, we explored the associations of the levels of Aβ and tau with SV2A and SYP in the hippocampus and entorhinal cortex of AD patients and NCs. 4G8, AT8, SV2A and SYP staining was performed on the adjunct slices of the tissue samples (Fig. 5). We found no associations between the CERAD-neuritic plaque score and the SV2A level or SYP level in the brains of AD patients and NCs. The correlation between SV2A and the 4G8 Aβ% area was not strong in the DG (*P* = 0.0122, *r* = − 0.3748), CA3/CA2 (*P* = 0.0384, *r* = − 0.3246), CA1 (*P* = 0.0307, *r* = − 0.3263) or the entorhinal cortex (*P* = 0.0051, *r* = − 0.4448) in the combined AD + NC cohort (Fig. 6a–d). Robust negative correlations between the SV2A level and Braak stage were observed in the AD + NC group in the DG (*P* = 0.0028, *r* = − 0.9643), CA3/CA2 (*P* = 0.0135, *r* = − 0.8581), CA1 (*P* = 0.0238, *r* = − 0.8571), and subiculum (*P* = 0.0123, *r* = − 0.8929) regions and in the entorhinal cortex (*P* = 0.0238, *r* = − 0.8571) (Fig. 6e–i, Table S2). In AD patients, NCs, and the AD + NC combined cohort, there was a strong positive correlation between SV2A and SYP in the hippocampus, entorhinal cortex and gray matter of the cortex (Fig. 6k–p). In addition, S V2A and AT8 (phospho-tau) levels were negatively correlated in the subiculum (*P* = 0.0326, *r* = − 0.4374) of AD patients (Fig. 6j, Table S2). In contrast, no correlation was detected between SYP and 4G8 or AT8 levels or between SYP and the CERAD score or Braak stage in AD patients or NCs (Table S2).

To further assess the associations between SV2A and different types/morphologies of Aβ and tau, double staining was performed for SV2A/6E10 and SV2A/AT8. A reduced level of SV2A was more evident in the cored plaque than in the diffuse plaque (Fig. 5c–f). Mature tangles, ghost tangles and neuritic plaques were associated with reduced levels of SV2A (Fig. 5g–n), whereas the presence of neuropil thread did not influence the intensity of SV2A staining (Fig. 5). These results indicate a close association of SV2A with Aβ and tau pathologies.

## Discussion

One of the main strengths of our study is the comprehensive characterization of synaptic markers in a relatively large cohort of postmortem brain tissues from AD patients and NCs. We performed proteomic analyses on BDEVs isolated from 17 AD patients and 4 NCs, while IHC was conducted on samples from 40 AD patients and 44 NCs. SV2A emerged as a central hub owing to its numerous interactions with proteins from subnetworks related to synaptic vesicle formation and fusion. The observed alterations in synaptic proteins and their associations with Aβ, tau, Braak staging, and the *APOE* ε4 allele provide postmortem validation for in vivo imaging and fluid biomarkers of synaptic density.

First, we detected a significant reduction in the BDEV levels of SV2A and other synaptic proteins, such as SYT1, SNAP25, and 14-3-3ζ, in AD patients compared with NCs. The strong correlations among these synaptic markers further reinforce their role in synaptic pathology. Interestingly, while some markers, such as GAP43 and SYT1, were already reduced at early Braak stages, others were significantly decreased only at later disease stages. These findings suggest a dynamic progression of synaptic degeneration in AD, warranting further analyses in larger cohorts covering different disease stages. Our findings align with previous studies reporting significantly lower levels of synaptic proteins in plasma-isolated neural-derived exosomes from AD patients, which were correlated with cognitive decline [54]. Similarly, recent investigations into CSF synaptic biomarkers identified alterations in 14-3-3 ζ/δ as promising pathophysiological changes in AD, whereas neuronal pentraxins were highlighted as general indicators of neurodegeneration across various dementias [53]. SV2A levels in both CSF and serum are positively correlated with cognitive performance and decrease as AD progresses [30, 55]. These findings collectively reinforce the value of SV2A as a potential biomarker of synaptic loss in AD. Additionally, we observed negative correlations between SV2A and BDEV levels of both GFAP and NEFL in AD patients. Synaptic biomarkers such as neurogranin, NEFL, and SNAP25 have been previously shown to be positively associated with CSF and plasma levels of GFAP in AD [34, 56]. A recent study also revealed a significant negative correlation between serum SV2A and GFAP concentrations in cognitively unimpaired individuals at risk of AD [55]. Moreover, PET imaging with [^18^F]SynVesT-1 demonstrated that brain SV2A levels are negatively associated with plasma GFAP and p-tau181 in cognitively impaired individuals [57]. Earlier studies have shown that astrocyte- and microglia-mediated synaptic loss contributes to the development of cognitive impairments in AD [16, 58]. Although we did not find significant differences in GFAP expression in the present BDEV proteomics dataset, studies using complementary methodologies (e.g., targeted immunoassays and flow cytometry) are currently underway in our laboratory. In addition, we have obtained a larger, better-matched dataset showing increased GFAP levels in AD-derived BDEVs (paper under review). While glial-mediated synaptic pruning has been implicated in AD, we did not directly quantify glial cell density, activation, or phagocytosis in our samples in this study. Thus, the hypothesis that astrocytes or microglia contribute to SV2A reduction remains speculative and warrants further investigation using glial-specific markers and functional assays. Future studies should explore glial contributions to synaptic marker clearance using approaches targeting specific cell types and quantification of glial activation.

Our proteomics and staining data both identified significant negative correlations between SV2A and p-tau, with a stronger correlation with p-tau than with Aβ. These findings provide valuable postmortem validation for in vivo PET imaging studies that investigate the relationships among SV2A, Aβ, and tau burden. PET studies have consistently reported negative associations between SV2A and tau using [^11^C]UCB-J with [^18^F]MK-6240 [71] or [^18^F]flortaucipir [59], and using [^18^F]SynVesT-1 with [^18^F]MK-6240 [60]. A recent study demonstrated that longitudinal synaptic loss is associated with tau pathology in patients with amnestic MCI and in cognitively impaired individuals [60, 61]. As pathological tau spreads transsynaptically in a ‘prion-like’ manner, the deposition of hyperphosphorylated tau at multiple points can consequently spread along axons and lead to pronounced synaptic loss in widespread regions. An earlier study using sub-diffraction-limit microscopy, demonstrated that synaptic oligomeric tau is likely the cause of the spread of tau pathology throughout the brain in AD patients [62]. In contrast to tau, results regarding SV2A and Aβ have been mixed: while some PET studies reported a "paradoxical" positive correlation between SV2A and [^11^C]PiB in the hippocampus of AD patients [63], others reported a negative correlation between [^18^F]SynVesT-1 and [^18^F]florbetapir in the hippocampus and parahippocampus [64]. In addition, Aβ oligomers, rather than plaques, have been shown to induce synaptic degeneration in AD animal models [65]. Our findings suggest that SV2A is more strongly associated with tau pathology than with Aβ deposition, supporting the hypothesis that tau accumulation and its synaptic spread may play critical roles in synaptic dysfunction in AD [62, 66].

Our IHC results revealed significantly reduced SV2A levels in the hippocampus and entorhinal cortex of AD patients, whereas no significant differences were detected in the frontal or temporal cortex. The hippocampus is particularly vulnerable to early synaptic loss because of the degeneration of entorhinal cortical neurons that project to this region via the perforant path [7, 67, 68]. Our results are consistent with previous reports indicating comparable SV2A expression levels in the middle frontal gyrus of AD patients and NCs via ELISA and Western blotting [69], whereas immunostaining studies (with smaller sample sizes of *n* = 6–7 per group) have reported reductions of both SV2A and SYP in the middle as well as inferior frontal gyrus of AD patients [70]. However, conflicting findings have emerged from studies using radiolabeled ligands, with some reporting decreased [69] and others unchanged [71] [^3^H]UCB-J binding in the middle frontal gyrus of AD patients. Similarly, autoradiography studies have reported unchanged [^3^H]UCB-J binding in the parietal, temporal, and occipital cortices of AD patients compared with NCs [69]. More recent immunoblotting studies have revealed reductions in [^3^H]UCB-J binding but no significant changes in SV2A protein levels in homogenates or synaptosome fractions from the frontal cortex, temporal cortex, or hippocampus of AD patients [72]. In contrast, in vivo PET imaging studies using various SV2A tracers have consistently reported decreased uptake in the hippocampus and temporal cortex of AD patients [3, 11, 22, 23, 29, 31, 69, 73], with conflicting results for the frontal cortex. These discrepancies between in vivo and postmortem findings could be attributed to methodological differences, including postmortem degradation effects, differences in tracer affinity, volumetric alterations, or glucose metabolism influences on in vivo SV2A uptake [74, 75].

*APOE* ε4 is the strongest genetic risk factor for sporadic AD and has been implicated in synaptic dysfunction in cellular and animal models [76]. Recent imaging studies have demonstrated that cognitively impaired individuals carrying *APOE* ε4 exhibit a decreased level of SV2A PET uptake and a lower serum level of SV2A compared to *APOE* ε4 non-carriers [55, 77]. In addition, cognitively unimpaired *APOE* ε4 homozygotes show reduced SV2A PET uptake in the hippocampus compared to *APOE* ε4 non-carriers [78]. Our postmortem findings corroborate these observations, as significantly lower hippocampal SV2A levels were detected in AD *APOE* ε4 carriers than in noncarriers. Studies in E3FAD and E4FAD mouse models suggest that *APOE* ε4 contributes to synaptic dysfunction through alterations of dendritic spine density and expression of synaptic proteins [79]. Furthermore, *APOE* ε4 exacerbates tau-mediated neurodegeneration in tauopathy models [80], whereas astrocytic APOE expression appears to play a key role in synaptic degeneration by promoting microglial phagocytosis of synaptic elements [81].

The relationship between SV2A and SYP remains incompletely understood. In our study, we observed a strong correlation between SV2A and SYP levels across both AD and NC brain tissue samples, which is consistent with our BDEV proteomic findings and previous reports [70]. However, studies in postmortem brain tissues of PD, PDD, and DLB have shown that, SV2A and SYP exhibit only weak correlations despite their shared presynaptic localization [82]. This discrepancy may be partially explained by individual variability in synaptic density [83] and disease-specific alterations in synaptic organization. Moreover, the laminar distributions of SV2A and SYP in our samples were generally homogeneous, with only subtle differences—supporting observations from previous studies [70, 82, 84, 85]. Taken together, the robust correlation between SV2A and SYP in both BDEVs and tissue samples reinforces the value of SV2A as a reliable synaptic marker in AD. Nonetheless, the divergent findings in other neurodegenerative disorders underscore the need for further research to clarify the distinct roles of presynaptic proteins across different disease contexts. Identifying more specific roles of synaptic biomarkers may separate synaptic biomarkers from the broader neurodegeneration biomarker category for AD [1, 86].

Although the sample size for BDEV proteomics was limited, all fresh-frozen postmortem samples were collected at CHUV and Lille using a single standardized protocol, which reduced the degree of technical variability. MS-based proteomics presents inherent limitations regarding batch effects: additional samples cannot be added ad hoc without reprocessing of the entire cohort, as changes in chromatographic columns or analytical instruments may bias the comparability. While the sample size for proteomic analyses was modest, it was counterbalanced by the high quality and uniform processing of the samples. Comparisons between BDEV and tissue-based findings must be interpreted cautiously, as different brain regions were analyzed due to the experimental constraints.

This study has several limitations. First, two separate groups of samples were analyzed. The cohort used for proteomic analysis was smaller and distinct from the group used for immunostaining. We were not able to obtain sufficient numbers of hippocampal samples to perform both proteomic and IHC analyses on the same synaptic preparation. We attempted IHC using postfixed frozen tissues from our available samples; however, the staining quality was not satisfactory. As a result, the tissue used for synaptic IHC and that used for proteomics came from different cases. Second, there was a sex imbalance within the AD group. Third, the samples used for proteomics and IHC differed in age and PMD. Fourth, we did not include antibodies for Aβ or tau oligomers (e.g., A11 or T22), which could have provided additional mechanistic insights. Fifth, we did not distinguish between early-onset and late-onset AD cases, which may follow different pathophysiological trajectories. Last, owing to the retrospective nature of sample collection, we lacked access to medical histories or MMSE scores—particularly near the time of death—which limited our ability to correlate SV2A levels with cognitive status.

In conclusion, this study revealed reduced regional levels of SV2A in brain tissues and BDEVs in AD patients than in NCs, particularly in those carrying the *APOE* ε4 allele, as well as correlations of SV2A with Aβ and tau pathologies. SV2A is a central hub in synaptic vesicle formation and fusion. These findings suggest that SV2A may serve as a valuable biomarker for synaptic dysfunction in AD, with potential utility in diagnostic panels and clinical trials targeting synaptic degeneration. Further research is needed to validate these biomarkers in longitudinal studies.

## Supplementary Information

A**dditional file 1.** **Table S1**. Antibodies and chemicals used for immunochemical/immunofluorescence. **Table S2**. Correlation analysis of hippocampal SV2A and synaptophysin in AD patients and NCs. **Fig. S1**. The postmortem delay (PMD) did not differ between AD and NCs, or affect the quality of EVs. **Fig. S2**. Representative images of H&E-stained images of the hippocampus, entorhinal cortex, frontal cortex and temporal cortex of AD patients and NCs. **Fig. S3**. Representative overview of 4G8 amyloid-β (brown) immunohistochemical staining in the frontal cortex, temporal cortex and temporal cortex of NC and AD groups. **Fig. S4**. Representative overview of AT-8 phospho-tau immunofluoresence staining in the hippocampus of NC and AD groups. **Fig. S5**. Braak stage does not influence the quantity or amount of isolated BDEVs in the prefrontal cortex of NC and AD cases. **Fig. S6**. Relative abundance of brain-specific proteins in different cellar subtypes of NC and AD patients of different Braak stages. **Fig. S7**. Comparison and correlation between cortical synaptosome BDEV markers in NC and AD. **Fig. S8**. Nonparametric Spearman rank analysis of the rIBAQ matrix of correlations in the AD and NC groups. **Fig. S9**. Nonparametric Spearman rank analysis of the rIBAQ matrix of correlation in the AD group. **Fig. S10**. Nonparametric Spearman rank analysis of SV2A with other BDEVs in the AD and NC groups. **Fig. S11**. SV2A staining in the hippocampus, frontal cortex and temporal cortex of NC and AD *APOE* ε4 carriers and noncarriers.

## Data Availability

The data are available upon reasonable request from the corresponding authors.

## Abbreviations

AD: Alzheimer’s disease
Aβ: Amyloid-β
APOE ε4: Apolipoprotein E ε4 allele
BDEVs: Brain-derived extracellular vesicles
CERAD: Consortium to establish a registry for Alzheimer’s disease
CSF: Cerebrospinal fluid
DG: Dentate gyrus
EVs: Extracellular vesicles
GAP43: Growth-associated protein 43
GFAP: Glial fibrillary acidic protein
H&E: Hematoxylin and eosin
IHC: Immunohistochemistry
LC‒MS/MS: Liquid chromatography tandem mass spectrometry
MCI: Mild cognitive impairment
MISEV: Minimal information for studies of extracellular vesicles
NBB: Netherlands Brain Bank
NCs: Nondemented controls
NEFL: Neurofilament light chain
PET: Positron emission tomography
PMD: Postmortem delay
SIGNOR: Signaling network open resources
SV2A: Synaptic vesicle protein 2A
SYP: Synaptophysin
SNAP25: Synaptosomal-associated protein 25 kDa
SYT1, 7: Synaptotagmin 1, 7:
TUBB3: Tubulin beta 3 class III
WGCNA: Weighted gene coexpression network analysis
