# Supplementary material for: Reduced synaptic vesicle protein 2A in extracellular vesicles and brains of Alzheimer’s disease: associations with Aβ, tau, synaptic proteins and APOE ε4
Source: Transl Neurodegener. 2025 Sep 24;14:48. doi: 10.1186/s40035-025-00508-2 (PMC12459057; doi:10.1186/s40035-025-00508-2)
Supplement: Supplementary file 1 — Additional file 1. Table S1. Antibodies and chemicals used for immunochemical/immunofluorescence. Table S2. Correlation analysis of hippocampal SV2A and synaptophysin in AD patients and NCs. Fig. S1. The postmortem delay (PMD) did not differ between AD and NCs, or affect the quality of EVs. Fig. S2. Representative images of H&E-stained images of the hippocampus, entorhinal cortex, frontal cortex and temporal cortex of AD patients and NCs. Fig. S3. Representative overview of 4G8 amyloid-β (brown) immunohistochemical staining in the frontal cortex, temporal cortex and temporal cortex of NC and AD groups. Fig. S4. Representative overview of AT-8 phospho-tau immunofluoresence staining in the hippocampus of NC and AD groups. Fig. S5. Braak stage does not influence the quantity or amount of isolated BDEVs in the prefrontal cortex of NC and AD cases. Fig. S6. Relative abundance of brain-specific proteins in different cellar subtypes of NC and AD patients of different Braak stages. Fig. S7. Comparison and correlation between cortical synaptosome BDEV markers in NC and AD. Fig. S8. Nonparametric Spearman rank analysis of the rIBAQ matrix of correlations in the AD and NC groups. Fig. S9. Nonparametric Spearman rank analysis of the rIBAQ matrix of correlation in the AD group. Fig. S10. Nonparametric Spearman rank analysis of SV2A with other BDEVs in the AD and NC groups. Fig. S11. SV2A staining in the hippocampus, frontal cortex and temporal cortex of NC and AD APOE ε4 carriers and noncarriers. [file 40035_2025_508_MOESM1_ESM.docx]

**Table S1. Antibodies and chemicals used for immunochemical/immunofluorescence staining**

| **Item** | **Catalog no** | **Dilution** | **Supplier** |
| --- | --- | --- | --- |
| Mouse phospho-Tau (Ser202, Thr205) monoclonal antibody (AT8) | MN1020 | 1:1000 | Invitrogen |
| Mouse purified anti-β-Amyloid, 17-24 monoclonal antibody (4G8, IHC) | 800701 | 1:4000 | Biolegend |
| Synaptophysin (27G12, IHC) | SYNAP-299-L-CE | 1:1000 | Leica Systems |
| Mouse purified anti-β-Amyloid, 1-16 monoclonal antibody (6E10, IF) | 803015 | 1:1000 | Biolegend |
| Anti-SV2A antibody (EPR23500-32, IHC, IF) | ab254351 | 1:1000 | Abcam |
| Alexa fluor488 donkey anti-mouse IgG (H+L) | 715-545-151 | 1:500 | Jackson ImmunoResearch |
| Alexa fluor674 donkey anti-rabbit IgG (H+L) | 711-605-152 | 1:500 | Jackson ImmunoResearch |
| Alexa fluor488 donkey anti-guinea pig IgG (H+L) | 706-545-148 | 1:500 | Jackson ImmunoResearch |
| Polymer Refine detection | DS9800 |  | Leica Systems |
| DAPI (4',6-Diamidino-2-Phenylindole, Dihydrochloride) | D1306 | 1:1000 | Invitrogen |

**Table S2. Correlation analysis of hippocampal SV2A and synaptophysin in AD patients and NCs**

|  | **Region** | **Group** | **SV2A** | | **SYP** | |
| --- | --- | --- | --- | --- | --- | --- |
|  |  |  | **r** | ***P* value** | **r** | ***P* value** |
| Braak Stage | CA1 | NC | 0.1000 | 0.9500 | 0.2000 | 0.9167 |
|  |  | AD | -0.3000 | 0.6833 | -0.4000 | 0.5167 |
|  |  | NC+AD | **-0.8571** | **0.0238** | -0.6000 | 0.2417 |
|  | CA2/3 | NC | -0.4919 | 0.3999 | 0.2000 | 0.9167 |
|  |  | AD | 0.0776 | 0.9100 | -0.4000 | 0.5167 |
|  |  | NC+AD | **-0.8581** | **0.0135** | -0.6571 | 0.1750 |
|  | DG | NC | 0.1000 | 0.9500 | 0.2000 | 0.9167 |
|  |  | AD | -0.4000 | 0.5167 | -0.4000 | 0.5167 |
|  |  | NC+AD | **-0.9643** | **0.0028** | -0.6000 | 0.2417 |
|  | SUB | NC | 0.6000 | 0.3500 | 0.2000 | 0.9167 |
|  |  | AD | -0.4000 | 0.5167 | -0.4000 | 0.5167 |
|  |  | NC+AD | **-0.8929** | **0.0123** | -0.6000 | 0.2417 |
|  | EC | NC | 0.4000 | 0.5167 | 1.0000 | 0.0833 |
|  |  | AD | -0.3000 | 0.6833 | -0.4000 | 0.5167 |
|  |  | NC+AD | **-0.8571** | **0.0238** | -0.7714 | :0.1028 |
| Tau (AT8) | CA1 | NC | 0.09474 | 0.6912 | -0.06708 | 0.7914 |
|  |  | AD | -0.1992 | 0.3397 | -0.2709 | 0.1903 |
|  |  | NC+AD | -0.02108 | 0.8907 | -0.2058 | 0.1854 |
|  | CA2/3 | NC | -0.04361 | 0.8551 | 0.1146 | 0.6508 |
|  |  | AD | -0.2654 | 0.1998 | -0.2577 | 0.2135 |
|  |  | NC+AD | -0.1337 | 0.3812 | -0.1549 | 0.3214 |
|  | DG | NC | 0.01053 | 0.9649 | -0.003096 | 0.9903 |
|  |  | AD | -0.2260 | 0.2670 | -0.01880 | 0.9274 |
|  |  | NC+AD | 0.09911 | 0.5123 | -0.05722 | 0.7122 |
|  | SUB | NC | 0.1386 | 0.5715 | -0.004902 | 0.9887 |
|  |  | AD | **-0.4374** | **0.0326** | -0.3122 | 0.1375 |
|  |  | NC+AD | -0.1808 | 0.2460 | -0.2016 | 0.2063 |
|  | EC | NC | **0.5539** | **0.0230** | 0.3250 | 0.2370 |
|  |  | AD | -0.1887 | 0.3884 | -0.02471 | 0.9109 |
|  |  | NC+AD | 0.1240 | 0.4458 | 0.06741 | 0.6876 |
| Aβ (4G8) | CA1 | NC | -0.2208 | 0.3362 | -0.1632 | 0.5045 |
|  |  | AD | 0.2905 | 0.1787 | 0.02429 | 0.9146 |
|  |  | NC+AD | **-0.3263** | **0.0307** | -0.2187 | 0.1696 |
|  | CA2/3 | NC | -0.3877 | 0.1010 | -0.3235 | 0.2050 |
|  |  | AD | 0.01186 | 0.9682 | -0.04677 | 0.8405 |
|  |  | NC+AD | **-0.3246** | **0.0384** | -0.1740 | 0.2962 |
|  | DG | NC | -0.3909 | 0.0797 | -0.08246 | 0.7372 |
|  |  | AD | 0.2421 | 0.2657 | -0.1779 | 0.4168 |
|  |  | NC+AD | **-0.3748** | **0.0122** | -0.6950 | 0.6950 |
|  | SUB | NC | -0.2501 | 0.2615 | -0.1489 | 0.5310 |
|  |  | AD | 0.09289 | 0.6734 | 0.06028 | 0.7847 |
|  |  | NC+AD | -0.2362 | 0.1182 | -0.1265 | 0.4187 |
|  | EC | NC | -0.3627 | 0.1529 | -0.2647 | 0.3207 |
|  |  | AD | -0.3988 | 0.0733 | :-0.2998 | 0.1990 |
|  |  | NC+AD | **-0.4448** | **0.0051** | -0.2934 | 0.0824 |
| SYP | CA1 | NC | **0.6843** | **0.0002** |  | |
|  |  | AD | **0.7685** | **<0.0001** |  |  |
|  |  | NC+AD | **0.7401** | **<0.0001** |  |  |
|  | CA2/3 | NC | **0.7730** | **<0.0001** |  |  |
|  |  | AD | **0.8030** | **<0.0001** |  |  |
|  |  | NC+AD | **0.7652** | **<0.0001** |  |  |
|  | DG | NC | **0.7826** | **<0.0001** |  |  |
|  |  | AD | **0.6876** | **0.0003** |  |  |
|  |  | NC+AD | **0.6660** | **<0.0001** |  |  |
|  | SUB | NC | **0.7362** | **<0.0001** |  |  |
|  |  | AD | **0.6896** | **0.0003** |  |  |
|  |  | NC+AD | **0.7560** | **<0.0001** |  |  |
|  | EC | NC | **0.6205** | **0.0027** |  |  |
|  |  | AD | **0.8330** | **<0.0001** |  |  |
|  |  | NC+AD | **0.7223** | **<0.0001** |  |  |

Analysis of immunohistochemical staining of in the hippocampus of 40 AD, 44 NC; AD, Alzheimer’s disease. NC, nondemented control group. Nonparametric Spearman’s rank correlation was used. r: Correlation coefficient. SYP, synaptophysin. No correlation between CERAD score with SV2A or synaptophysin was detected.


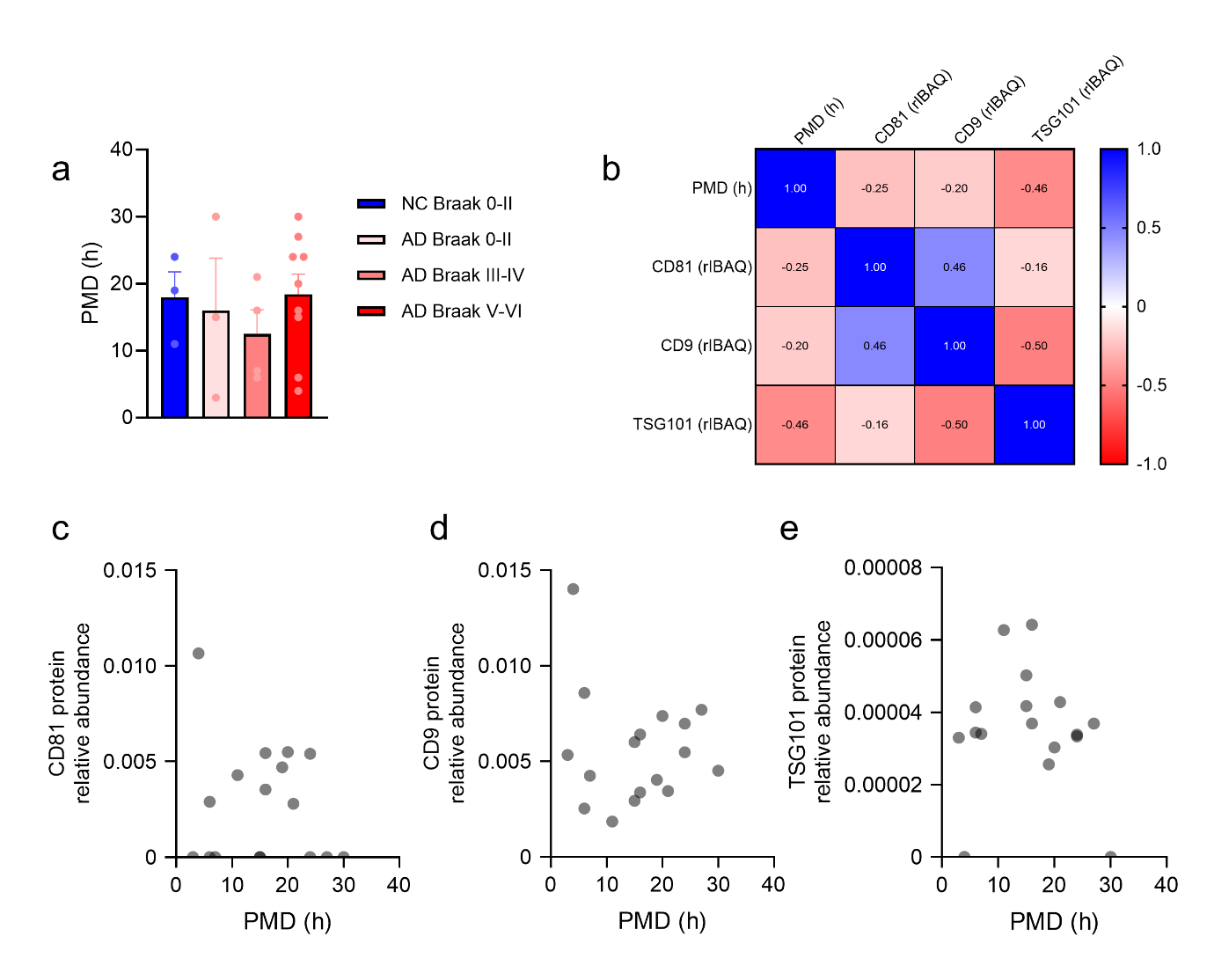


**Fig. S1** **The postmortem delay (PMD) did not differ between AD and NCs, or affect the quality of EVs.** **a** no difference between the PMD between NCs and AD cases of different Braak stages. **b-e** Heatmap and correlation plot (**b**) between the levels of (**c**) CD81, (**d**) CD9, and (**e**) TSG101 in BdEVs and the PMD of the tissues used. CD, cluster of differentiation; TSG101, tumor susceptibility gene 101 protein.


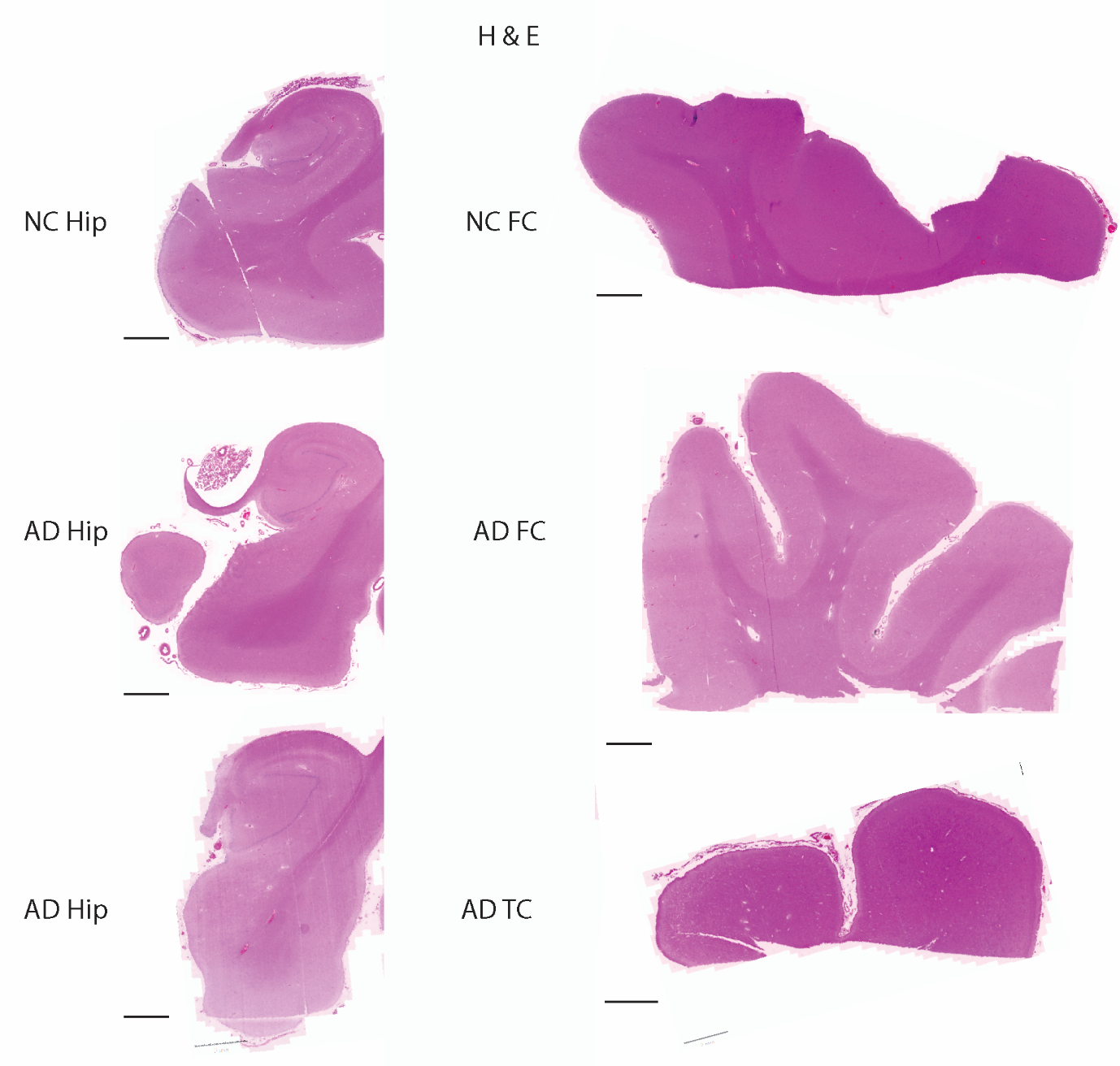


**Fig. S2 Representative images of H&E-stained images of the hippocampus, entorhinal cortex, frontal cortex and temporal cortex of AD patients and NCs.** Hip: hippocampus; H&E, hematoxylin and eosin; FC: frontal cortex; TC: temporal cortex. Scale bar, 2 mm.


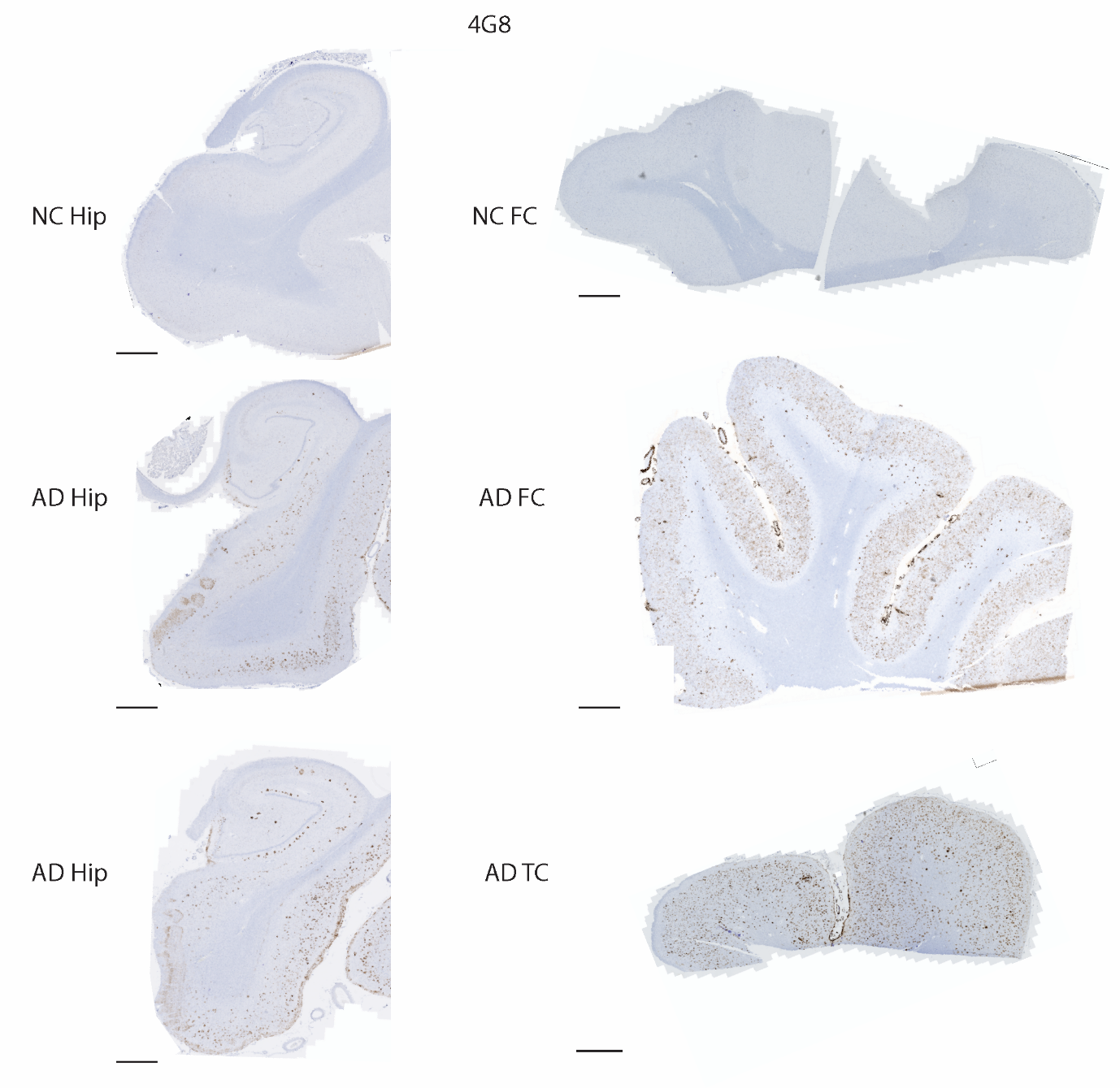
**Fig. S3 Representative overview of 4G8 amyloid-β (brown) immunohistochemical staining in the frontal cortex, temporal cortex and temporal cortex of NC and AD groups.** Hip: hippocampus; FC: frontal cortex; TC: temporal cortex. Scale bar, 2 mm.


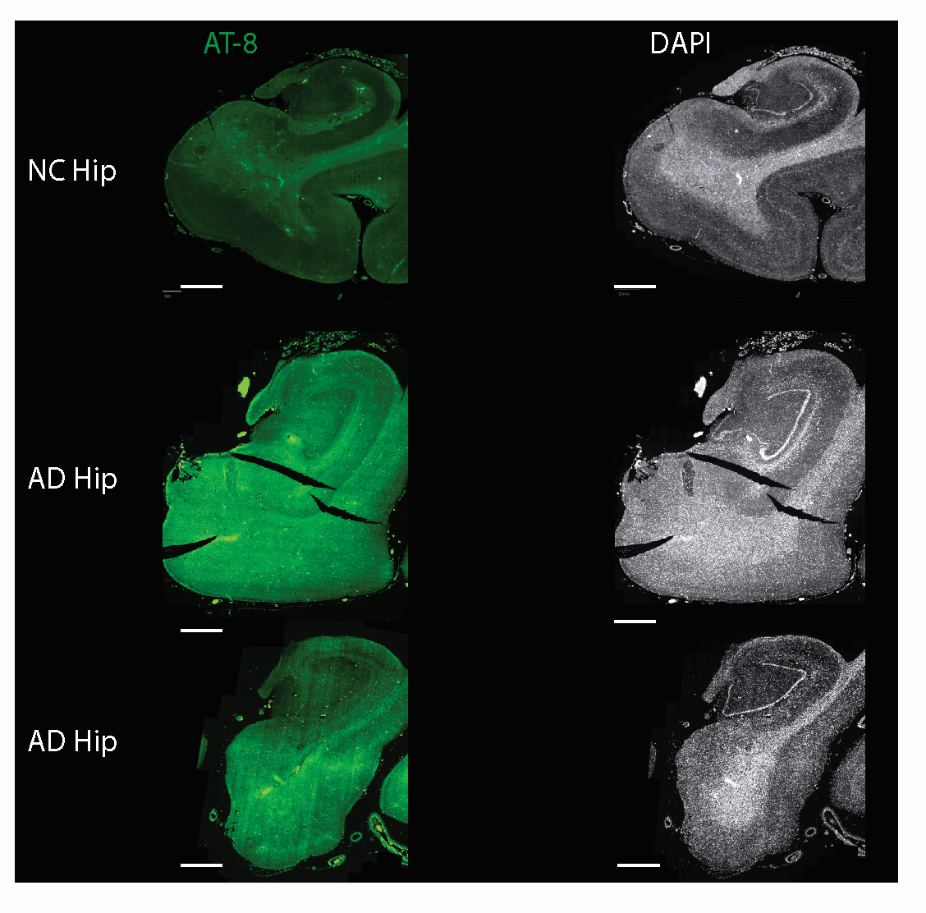


**Fig. S4** **Representative overview of AT-8 phospho-tau immunofluoresence staining in the hippocampus of NC and AD groups.** AT-8 (green). Nuclei was conterstained by DAPI. Hip: hippocampus; FC: frontal cortex; TC: temporal cortex. Scale bar, 2 mm.


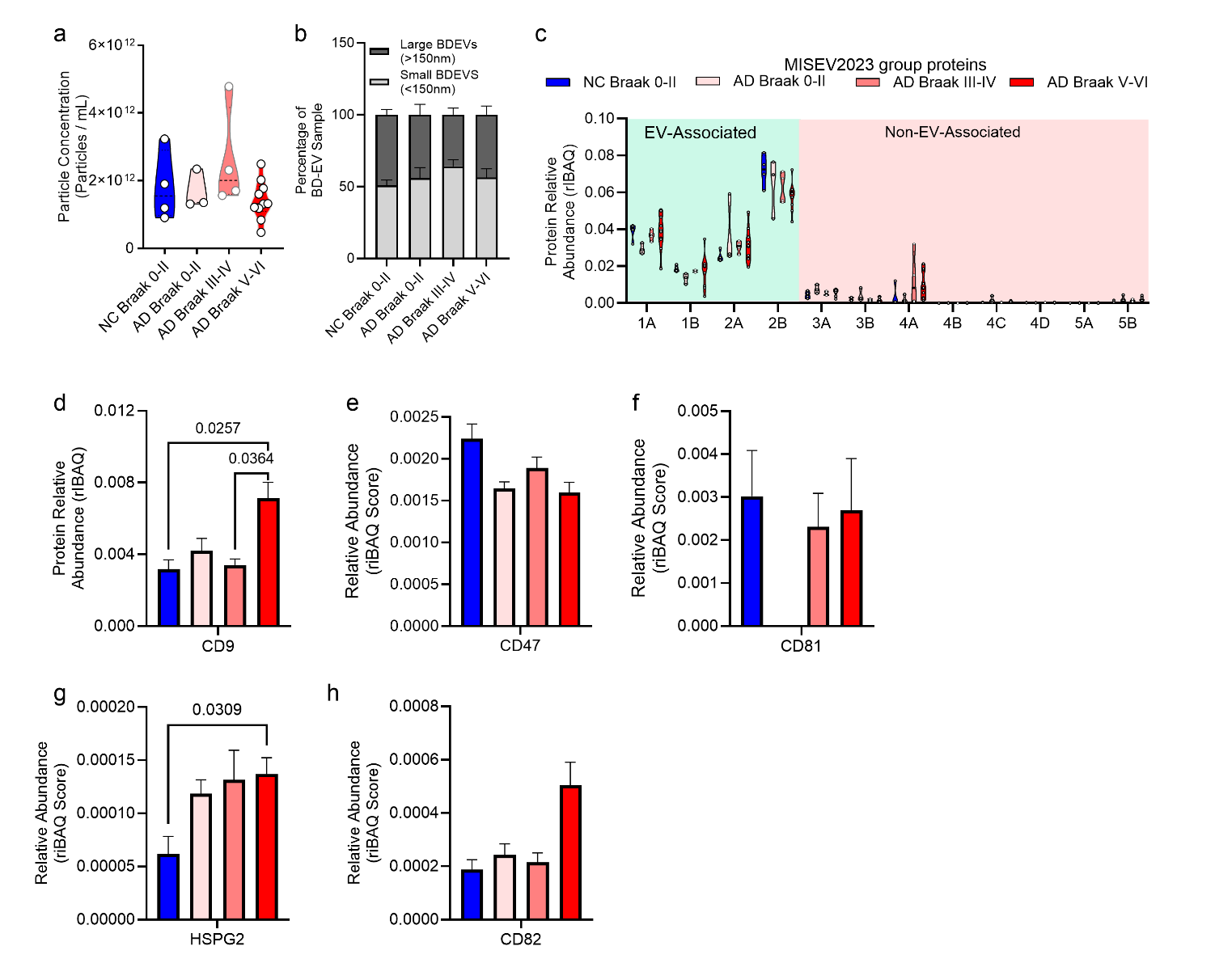
**Fig. S5 Braak stage does not influence the quantity or amount of isolated BdEVs in the prefrontal cortex of NC and AD cases. a** Histogram showing the number of BdEVs quantified by nanoparticle tracking analysis; **b** Stacked bar graph displaying the ratio of small (< 150 nm) to large (> 150 nm) EVs by patient group; **c-h** Bar graph quantifying the abundance of EV-associated proteins versus potentially contaminating proteins according to the MISEV2023 guideline categories; Histogram showing the relative quantity of different proteins in categories 1A and 1B, such as (**d**) CD9, (**e**) CD47, (**f**) CD81, (**g**) HSPG2, and (**h**) CD82. CD, cluster of differentiation; HSPG2, heparan sulfate proteoglycan 2;


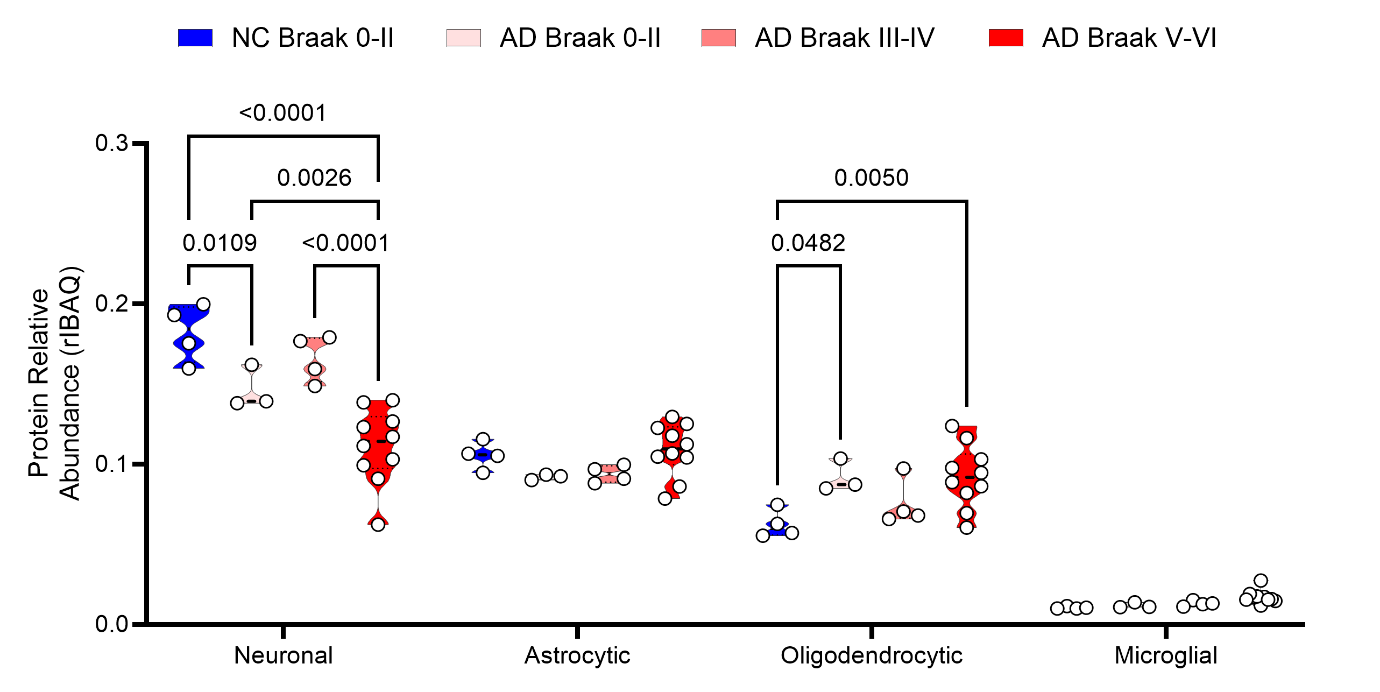


**Fig. S6** **Relative abundance of brain-specific proteins in different cellar subtypes of NC and AD patients of different Braak stages**. The major subtypes shown are neuronal, astrocytic, oligodendrocytic, and microglial cells.


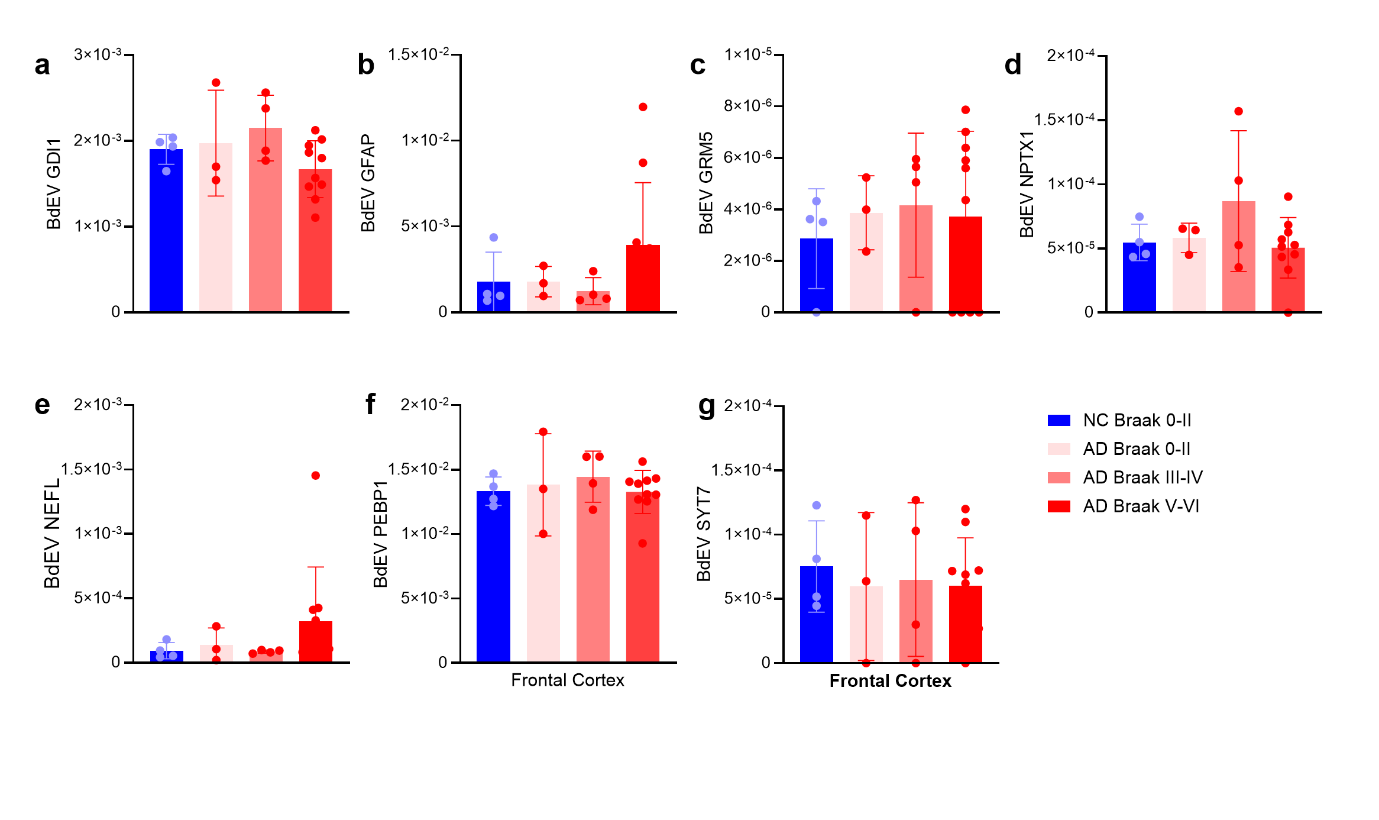


**Fig. S7 Comparison and correlation between cortical synaptosome BdEV markers in NC and AD a-g** Comparison of BdEV levels of GDI1, GFAP, GRM5, NPTX1, NEFL, PEBP1, and SYT7 in the NC Braak 0-II, AD of Braak 0-II, III-IV, and V-VI.

**
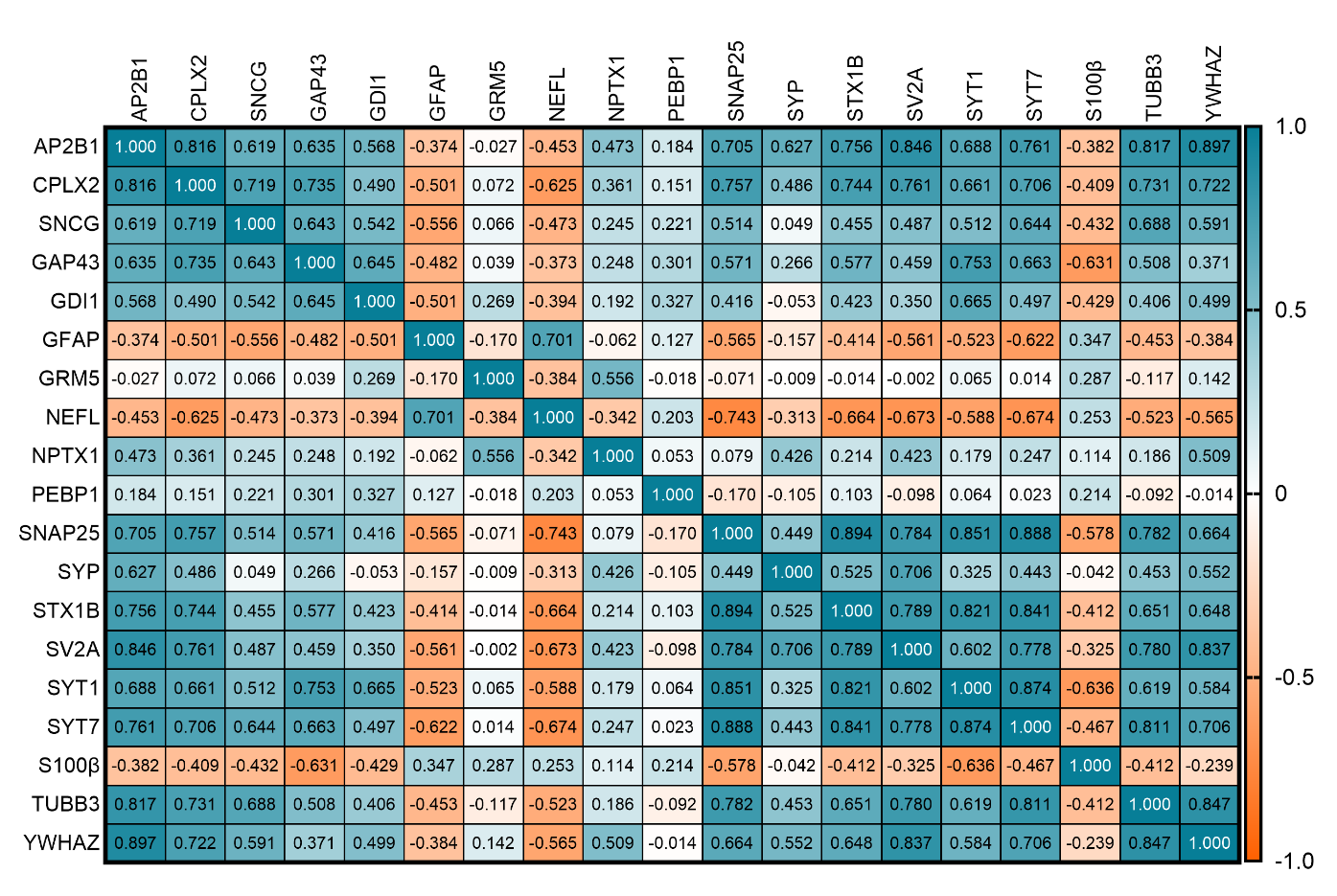
**

**Fig. S8 Nonparametric Spearman rank analysis of the rIBAQ matrix of correlations in the AD and NC groups.** The value in each cell indicates the correlation index.


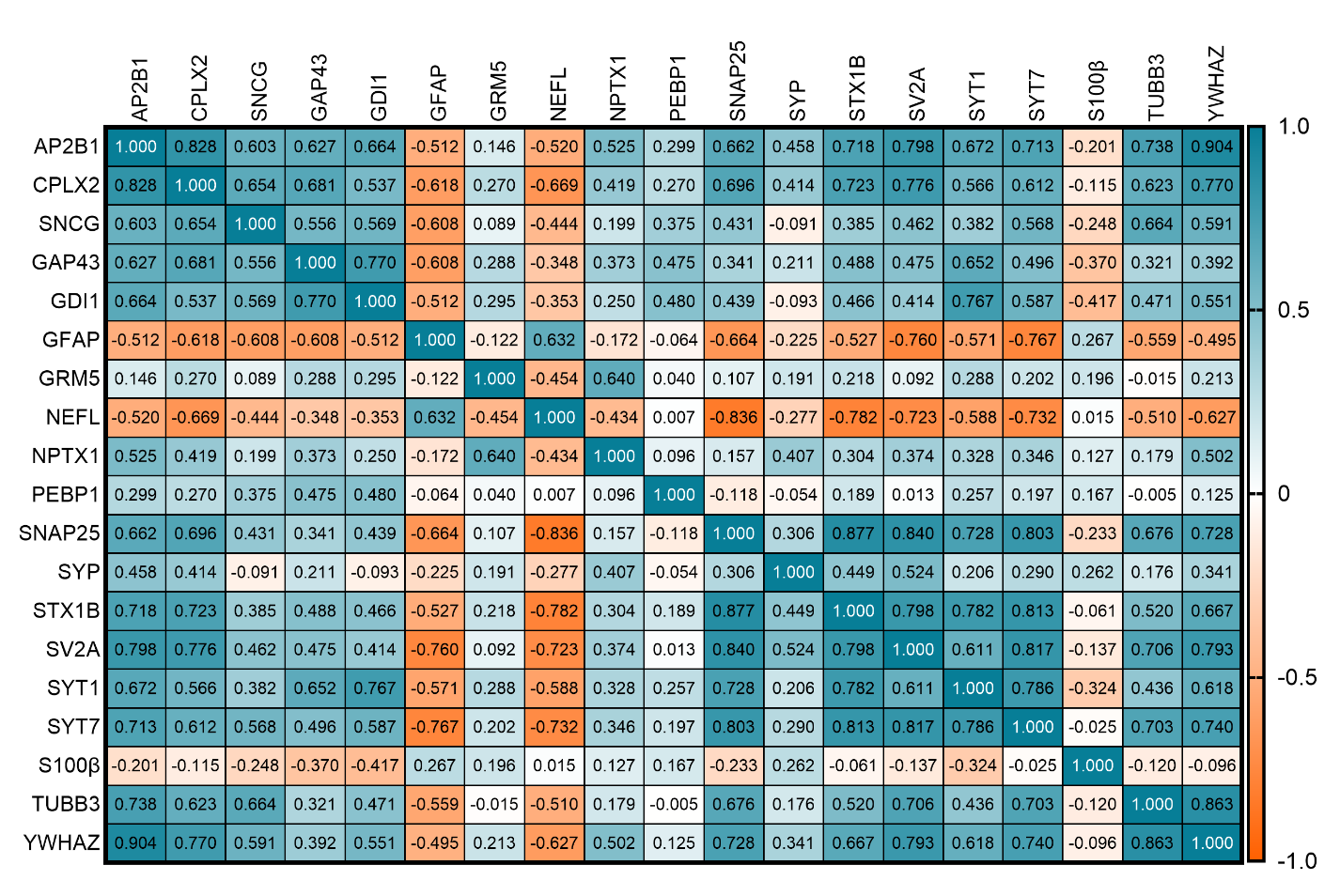
**Fig. S9 Nonparametric Spearman rank analysis of the rIBAQ matrix of correlation in the AD group**. The values in each cell indicate the correlation index.


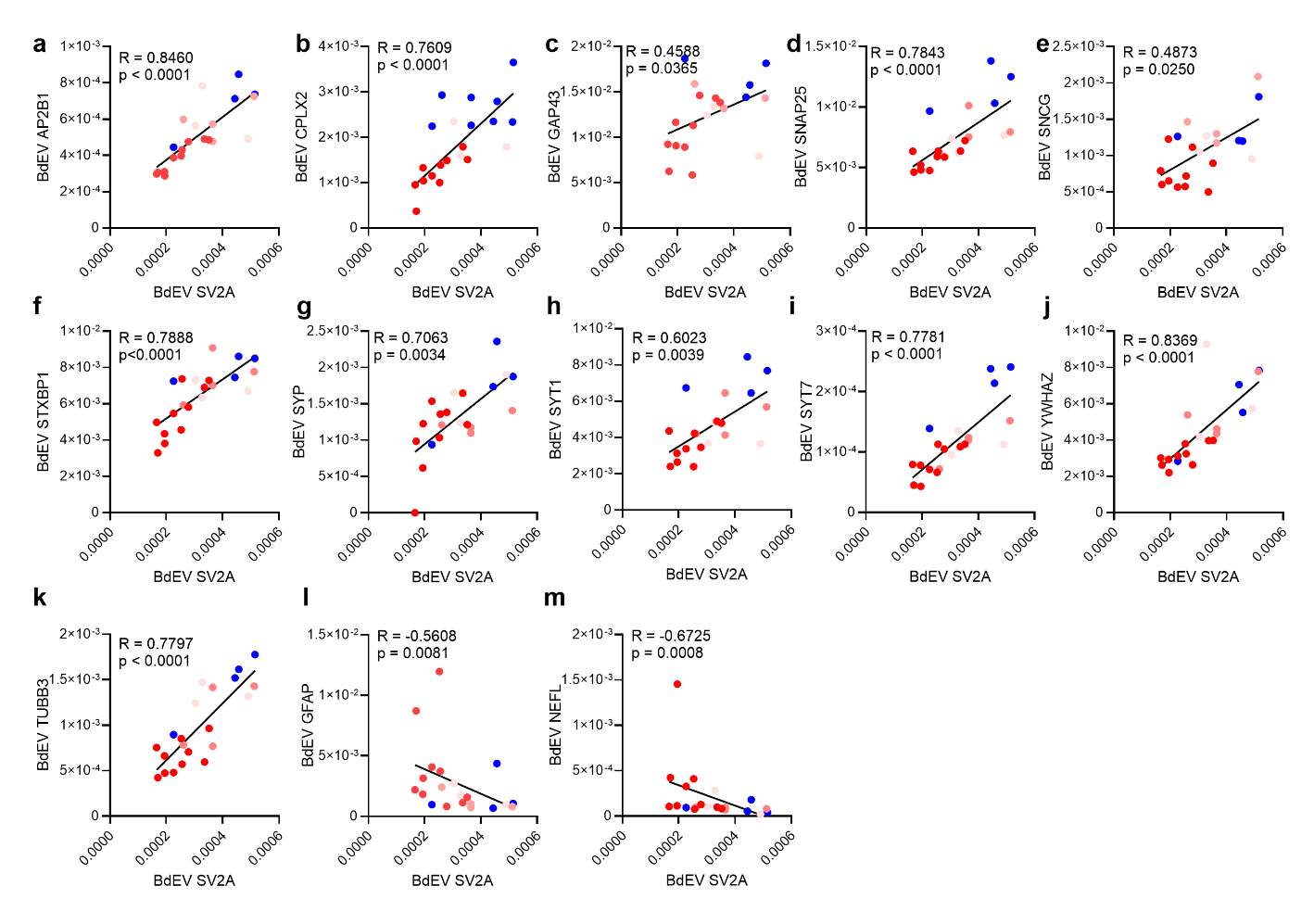


**Fig. S10 Nonparametric Spearman rank analysis of SV2A with other BdEVs in the AD and NC groups a-m** AP2B1, CPLX2, GAP43, SNAP25, SNCG, STX1B, SYP, SYT1, SVT7, YWHAZ, TUBB3, GFAP, and NEFL.


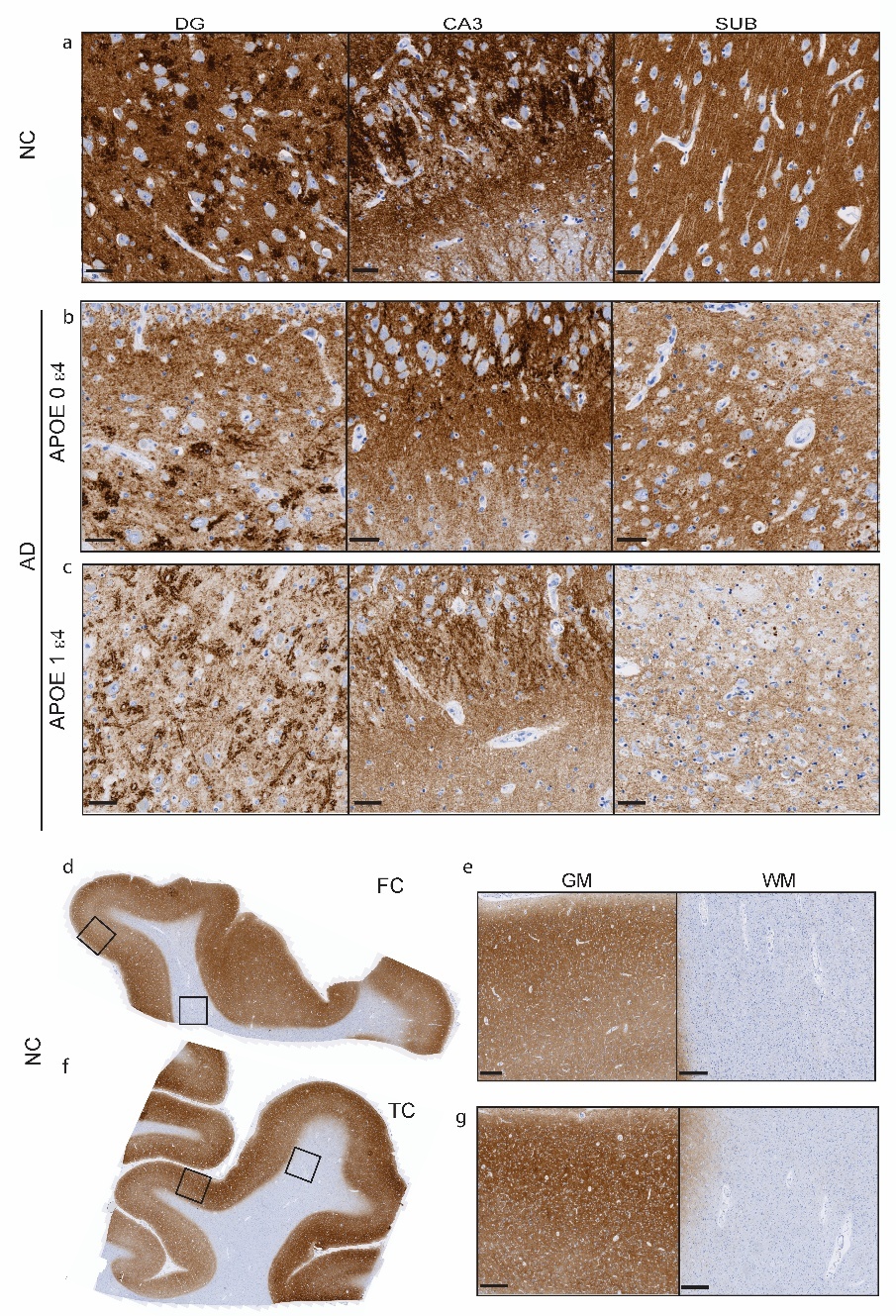


**Fig. S11 SV2A staining in the hippocampus, frontal cortex and temporal cortex of NC and AD *APOE* ε4 carriers and noncarriers. a-c** Images of immunohistochemical staining for SV2A in the hippocampi of NC and AD APOE ε4 noncarriers and carriers. **d-g** Representative images of immunohistochemical staining for SV2A in the frontal cortex (FC) and temporal cortex (TC) of the NC group. Zoom-in images of gray matter (GM) and white matter (WM). Scale bars, 50 μm (a-c) and 400 μm (e, g).
